# Blood DNA methylation sites predict death risk in a longitudinal study of 12,300 individuals

**DOI:** 10.18632/aging.103408

**Published:** 2020-07-22

**Authors:** Elena Colicino, Riccardo Marioni, Cavin Ward-Caviness, Rahul Gondalia, Weihua Guan, Brian Chen, Pei-Chien Tsai, Tianxiao Huan, Gao Xu, Agha Golareh, Joel Schwartz, Pantel Vokonas, Allan Just, John M. Starr, Allan F. McRae, Naomi R. Wray, Peter M. Visscher, Jan Bressler, Wen Zhang, Toshiko Tanaka, Ann Zenobia Moore, Luke C. Pilling, Guosheng Zhang, James D. Stewart, Yun Li, Lifang Hou, Juan Castillo-Fernandez, Tim Spector, Douglas P. Kiel, Joanne M. Murabito, Chunyu Liu, Mike Mendelson, Tim Assimes, Devin Absher, Phil S. Tsaho, Ake T. Lu, Luigi Ferrucci, Rory Wilson, Melanie Waldenberger, Holger Prokisch, Stefania Bandinelli, Jordana T. Bell, Daniel Levy, Ian J. Deary, Steve Horvath, Jim Pankow, Annette Peters, Eric A. Whitsel, Andrea Baccarelli

**Affiliations:** 1 Icahn School of Medicine at Mount Sinai, New York, NY 10029, USA; 2 Centre for Genomic and Experimental Medicine, Institute of Genetics and Molecular Medicine, University of Edinburgh, Edinburgh EH4 2XU, UK; 3 US Environmental Protection Agency, Chapel Hill, NC 27514, USA; 4 Gillings School of Global Public Health, University of North Carolina, Chapel Hill, NC 27514, USA; 5 Division of Biostatistics, School of Public Health, University of Minnesota, Minneapolis, MN 55455, USA; 6 Longitudinal Study Section, Translational Gerontology Branch, National Institute of Aging, Bethesda, MD 20892, USA; 7 Department of Twin Research and Genetic Epidemiology, King’s College London, London SE1 7EH, UK; 8 National Heart, Lung, and Blood Institute, Bethesda, MD 20892, USA; 9 Columbia University Mailman School of Public Health, New York, NY 10032, USA; 10 Harvard T.H. Chan School of Public Health, Boston, MA 02115, USA; 11 VA Boston Healthcare System and Boston University Schools of Public Health and Medicine, Boston, MA 02115, USA; 12 Alzheimer Scotland Dementia Research Centre, University of Edinburgh, Edinburgh EH8 9JZ, UK; 13 Institute for Molecular Bioscience, University of Queensland, Brisbane, QLD, Australia; 14 University of Texas Health Science Center at Houston, Houston, TX 77030, USA; 15 Department of Biostatistics and Data Science, School of Public Health, University of Texas Health Science Center at Houston, Houston, TX 77030, USA; 16 Epidemiology and Public Health Group, University of Exeter Medical School, Exeter, UK; 17 Department of Genetics, University of North Carolina, Chapel Hill, NC 27514, USA; 18 Feinberg School of Medicine, Northwestern University, Chicago, IL 60611, USA; 19 Hebrew SeniorLife Institute for Aging Research and Department of Medicine, Beth Israel Deaconess Medical Center and Harvard Medical School Boston, MA 02215, USA; 20 Section General Internal Medicine, Department of Medicine, Boston University School of Medicine, Boston, MA 02215, USA; 21 Boston University School of Public Health, Boston, MA 02215, USA; 22 Boston University School of Medicine, Boston, MA 02215, USA; 23 Stanford University School of Medicine, Stanford, CA 94305, USA; 24 Hudson Alpha Institute for Biotechnology, Huntsville, AL 35806, USA; 25 Department of Human Genetics, David Geffen School of Medicine, University of California Los Angeles, Los Angeles, CA 90095, USA; 26 National Institute of Aging, Bethesda, MD 20892, USA; 27 Research Unit of Molecular Epidemiology, Helmholtz Zentrum Munich, German Research Center for Environmental Health, Neuherberg D-85764, Germany; 28 Institute of Human Genetics, Helmholtz Zentrum Munich, German Research Center for Environmental Health, Neuherberg S-85764, Germany; 29 Geriatric Unit, Azienda Sanitaria Firenze, Florence, Italy; 30 Framingham Heart Study, Framingham, MA 01702, USA; 31 Department of Psychology, University of Edinburgh, Edinburgh EH8 9JZ, UK; 32 Division of Epidemiology and Community Health, School of Public Health, University of Minnesota, Minneapolis, MN 55455, USA; 33 Institute for Epidemiology II, Helmholtz Zentrum Munich, German Research Center for Environmental Health, Neuherberg D-85764, Germany

**Keywords:** DNA methylation, 450K, all-cause mortality, epigenome-wide association studies, aging

## Abstract

DNA methylation has fundamental roles in gene programming and aging that may help predict mortality. However, no large-scale study has investigated whether site-specific DNA methylation predicts all-cause mortality. We used the Illumina-HumanMethylation450-BeadChip to identify blood DNA methylation sites associated with all-cause mortality for 12, 300 participants in 12 Cohorts of the Heart and Aging Research in Genetic Epidemiology (CHARGE) Consortium. Over an average 10-year follow-up, there were 2,561 deaths across the cohorts. Nine sites mapping to three intergenic and six gene-specific regions were associated with mortality (*P* < 9.3x10^-7^) independently of age and other mortality predictors. Six sites (cg14866069, cg23666362, cg20045320, cg07839457, cg07677157, cg09615688)—mapping respectively to *BMPR1B, MIR1973, IFITM3, NLRC5*, and two intergenic regions—were associated with reduced mortality risk. The remaining three sites (cg17086398, cg12619262, cg18424841)—mapping respectively to *SERINC2, CHST12*, and an intergenic region—were associated with increased mortality risk. DNA methylation at each site predicted 5%–15% of all deaths. We also assessed the causal association of those sites to age-related chronic diseases by using Mendelian randomization, identifying weak causal relationship between cg18424841 and cg09615688 with coronary heart disease. Of the nine sites, three (cg20045320, cg07839457, cg07677157) were associated with lower incidence of heart disease risk and two (cg20045320, cg07839457) with smoking and inflammation in prior CHARGE analyses. Methylation of cg20045320, cg07839457, and cg17086398 was associated with decreased expression of nearby genes (*IFITM3, IRF, NLRC5, MT1, MT2, MARCKSL1*) linked to immune responses and cardiometabolic diseases. These sites may serve as useful clinical tools for mortality risk assessment and preventative care.

## INTRODUCTION

The human epigenome contains DNA methylation marks that progressively change as we age. DNA methylation can influence gene expression and manifests in response to both environmental and hereditary factors [1, 2]. Biological age estimations, constructed from DNA methylation marks and referred to as “epigenetic aging clocks”, have been associated with environmental exposures, morbidities, and mortality [9–13]. As these clocks were designed to track chronological age, not to predict mortality, further study is necessary to fully elucidate indicators of all-cause mortality. To date, no large-scale analysis has been conducted to identify variations in DNA methylation at individual 5’-cytosine-phosphate-guanosine-3’ (CpG) sites associated with future mortality risk. Here, we present an epigenome-wide methylation analysis of 12,300 participants and 2, 561 (21%) deaths from 12 American and European cohorts to determine whether site-specific DNA methylation predicts all-cause mortality, independent of age, lifestyle factors, and clinical predictors of mortality including comorbidities. We also assessed the causal relationship of identified sites with age-related chronic diseases using Mendelian randomization approaches, and we related the sites to epigenetic aging clocks and a mortality risk score, an epigenetic indicator of mortality previously created and validated with DNA methylation arrays in two European cohorts.

## RESULTS

### Cohorts

**Figure 1:**
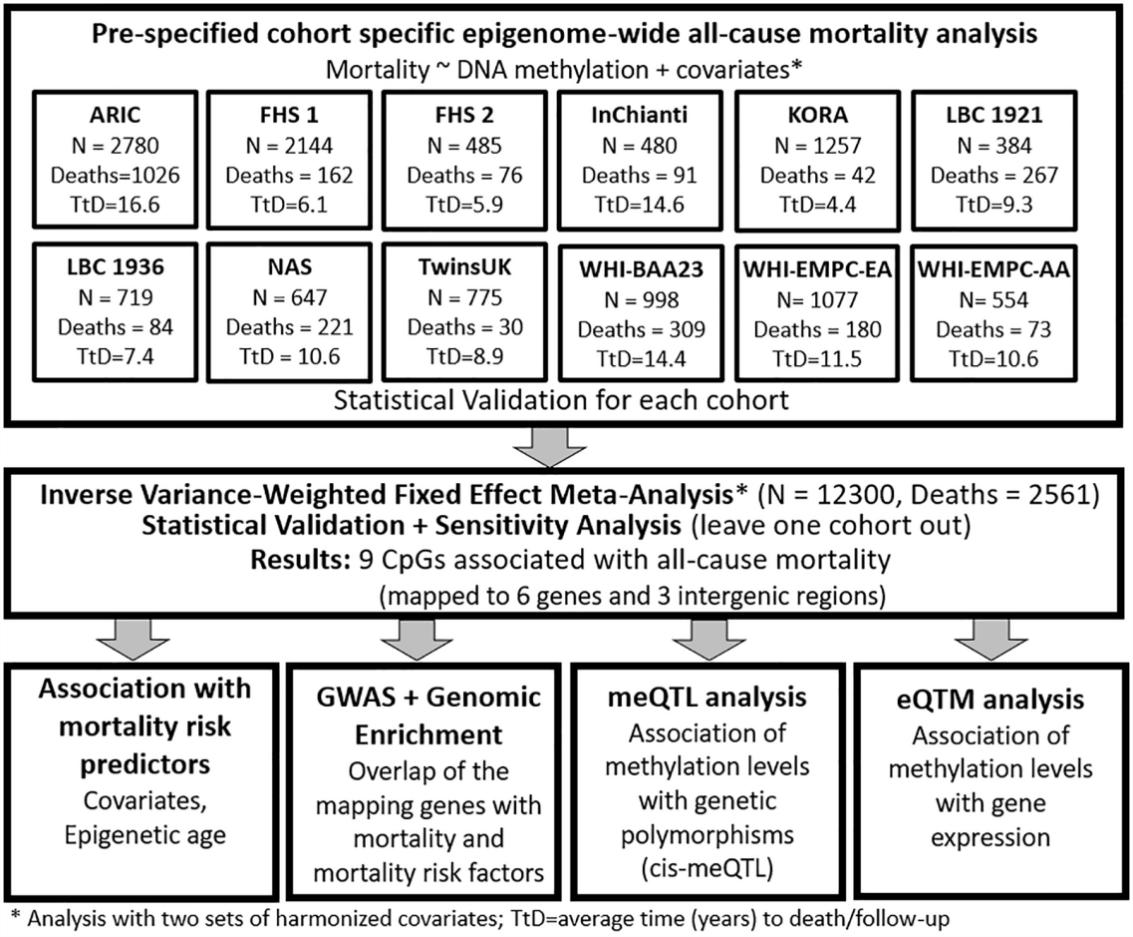
**Workflow of the study.**

Across studies in the Cohorts of the Heart and Aging Research in Genetic Epidemiology (CHARGE) Consortium, mortality rates ranged from 3%–70% of all participants, and the average time to death or censoring ranged from 4.4–16.6 years (Supplementary Table 1). Each study conducted epigenome-wide mortality analyses, adjusting for two sets of harmonized risk factors and confounders, and shared results for meta-analysis (Figure 1).

### Meta-analysis

**Figure 2:**
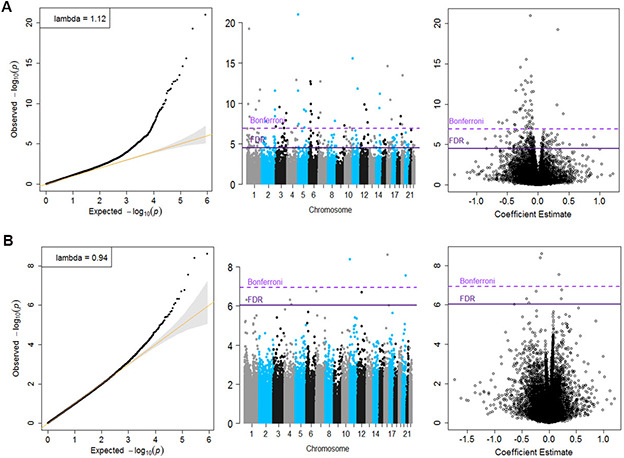
**Quantile-Quantile plots, Manhattan and Volcano for the basic model (Panel A) and for the fully adjusted model (Panel B).**

**Figure 3:**
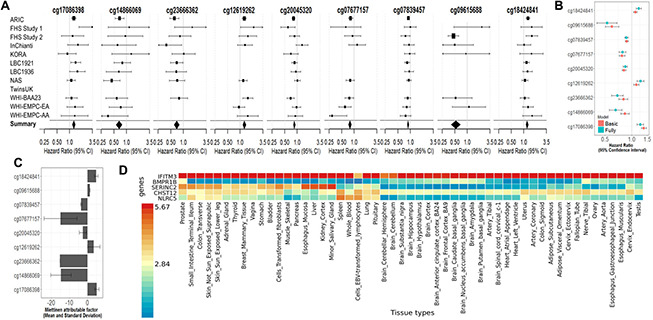
(**A**) Forest Plots for the association of methylation levels of the FDR-significant fully-adjusted CpGs with risk of all-cause mortality in the CHARGE consortium. (**B**) Sensitivity analysis. Comparison of the hazard ratio of the basic-adjusted and the fully-adjusted fixed effect meta-analysis. (**C**) Attributable factor. Predicted Contribution (%) of increased methylation levels (above the mean) of each CpG to the all-cause mortality associations in NAS, WHI-EMPC (EA) and WHI-EMPC (AA). (**D**) Functional Mapping and Annotation results in order to examine tissue specificity of the genes mapped to the FDR-significant fully-adjusted CpGs.

Inverse variance-weighted fixed-effects meta-analysis of 426, 724 CpGs identified 51 Bonferroni-significant and 257 FDR-significant (*P* < 3.03x10^-5^) CpGs in a basic model adjusting for age, sex, technical covariates, and white blood cell proportions (Figure 2A and Supplementary Table 2). We also identified three Bonferroni-significant and nine FDR-significant (*P* < 9.3x10^-7^) CpGs in a fully-adjusted model also adjusting for education, smoking status, pack-years smoked, body mass index, recreational physical activity, alcohol consumption, hypertension, diabetes, and history of cancer and coronary heart disease (Figures 2B, 3A and Supplementary Table 3). For 188 (73%) basic-adjusted FDR-significant CpGs and six (67%) fully-adjusted CpGs, higher blood DNA methylation was associated with lower all-cause mortality (Figure 2 and Supplementary Tables 2, 3).

All nine fully-adjusted FDR-significant CpGs had similar magnitude of associations with mortality in the basic model, although only five were also FDR-significant in the basic model (Figure 3B). Hazard ratios (HRs) of the nine fully-adjusted FDR-significant CpGs ranged 0.53–1.26 per 10% increase in DNA methylation levels, where 1 represents 100% methylation (Supplementary Table 4). Six sites (cg14866069, cg23666362, cg20045320, cg07839457, cg07677157, cg09615688) were associated with reduced mortality risk, while the remaining three sites (cg17086398, cg12619262, cg18424841) were associated with increased mortality risk (Figure 3A and Supplementary Tables 3, 4). Three fully-adjusted CpGs (cg07677157, cg09615688, cg18424841) were in intergenic regions; the remaining six (cg17086398, cg14866069, cg23666362, cg12619262, cg20045320, cg07839457) were within 10,000 bp of a gene, with two CpGs (cg07839457, cg23666362) mapped respectively to nucleotide-binding oligomerization domain-like receptor caspase recruitment domain containing 5 *(NLRC5)* and microRNA 1973 (*MIR1973)* within 1,500 bp of transcription start sites, and one (cg17086398) in the serine incorporator 2 (*SERINC2*) gene body (Supplementary Table 3).

Meta-analysis results did not appear to suffer from systematic bias due to unmeasured confounding, as assessed by genomic inflation (basic model: λ = 1.12, fully adjusted model λ = 0.94, Figure 2 and Supplementary Table 5). Cohort-specific inflation was also minimal, with lambdas close to one for most cohorts. Volcano plots showed symmetry in the direction of the associations with all-cause mortality (Figure 2). All nine fully-adjusted FDR-significant CpGs showed low/medium heterogeneity (Supplementary Table 7) and consistent magnitude of the estimated HRs across studies (Figure 3A). We further validated our results by excluding cohorts with high proportion of deaths (30%) and inflation (λ > 1.5). In these sensitivity analyses, HRs for the nine FDR-significant CpGs were consistent with main results in terms of direction, magnitude, and statistical significance (Supplementary Figure 1 and Supplementary Tables 8, 9).

**Figure 4:**
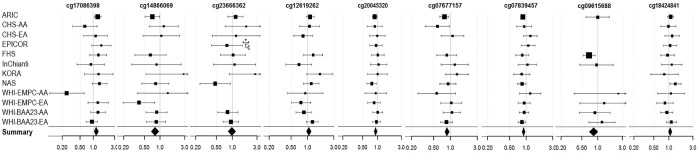
**Forest Plots for the association of methylation levels of the FDR-significant fully-adjusted CpGs with risk of future incident coronary heart disease in the CHARGE consortium.**

Three of the nine fully-adjusted FDR-significant CpGs (cg20045320, cg07677157, cg07839457) were associated with lower incidence of coronary heart disease rates (*P* < Bonferroni threshold of 0.005) (Figure 4 and Supplementary Table 10).

### Miettinen’s population attributable factor, epigenetic aging clocks, and mortality risk score

To assess the extent that methylation levels of each CpG predict all-cause mortality, we calculated Miettinen’s population attributable fraction on data from the Normative Aging Study (NAS) and the Women Health Initiative-Epigenetic Mechanisms of Particulate Matter-Mediated Cardiovascular Disease (WHI-EMPC) for European and African American ancestries. DNA methylation levels above the average at each CpG predicted, individually and independently of other factors, 5%–15% of all deaths (Figure 3C and Supplementary Table 11). In the same datasets, all nine CpGs were associated with age, cumulative smoking, body mass index, and physical activity (*P* < 0.05). Seven out of nine CpGs (cg17086398, cg14866069, cg23666362, cg20045320, cg7677157, cg07839457, cg09615688) had negative relationships with age (Supplementary Table 12). Seven CpGs were strongly associated with epigenetic aging clocks and mortality risk scores; all significant associations had the same direction and similar magnitude across the four epigenetic aging clocks (Supplementary Table 13), even if none of those sites was included in any of the clocks. Those CpGs had consistent and independent association with all-cause mortality when adjusted for epigenetic aging clocks and mortality risk scores (Supplementary Tables 14, 15). In overall meta-analysis, we identified 57 out of 58 CpGs of the risk score, and those sites had low to moderate association with DNA methylation levels at our FDR-significant CpGs with a balance between positive and negative correlations (Supplementary Figure 2A, 2B). In overall meta-analysis, the association between all-cause mortality and DNA methylation levels at the majority (34 out of 58) of mortality risk score CpGs had consistent direction with previous results. Among those CpGs, only two (cg25193885 and cg19859270) showed nominally significant association with mortality (Supplementary Figure 2A, 2B).

### Pathways analyses and DNA methylation integration with quantitative trait loci analysis (meQTL) and with gene expression (eQTM)

Extended genome-wide enrichment analysis showed that two of the CpGs (cg07839457 and cg17086398) mapped to genes (*NLRC5* and *SERINC2,* respectively) previously associated with high-density lipoprotein cholesterol levels (FDR = 0.02) and alcohol dependence (FDR = 0.004) in genome-wide association studies (GWAS) analyses (Supplementary Table 16) [14]. We confirmed these results using Database for Annotation, Visualization and Integrated Discovery (DAVID) and KEGG, identifying and testing for enriched underlying biological processes in publicly available gene ontology databases (Supplementary Tables 17, 18).

To characterize the functional relevance of FDR-significant CpGs, we performed covariate-adjusted methylation quantitative trait locus (meQTL) analyses using available unique single-nucleotide-polymorphism (SNP)–CpG combinations from 713 participants in the Cooperative Health Research in the Region Augsburg (KORA) study [15]. We identified nine Bonferroni-significant unique cis-regulatory polymorphisms associated with two 1000 bp-distant CpGs (cg09615688, cg18424841) (Supplementary Figure 3A and Supplementary Table 19). None of the nine identified polymorphisms overlapped with previous genetic results from the National Human Genome Research Institute-EBI GWAS Catalog (Supplementary Table 16).

We also evaluated expression quantitative trait methylation (eQTM) associations using 998 KORA participants. We identified three CpGs with FDR-significant associations with decreased leukocyte expression levels of nearby genes, among the 13, 351 unique associations between gene-expression and DNA methylation levels at FDR-significant fully-adjusted CpGs. Namely, DNA methylation levels of cg07839457 (in *NLRC5*) were associated with *NLRC5* expression as well as with that of a ~300 Mb-distant set of *metallothionein* (*MT*) *1* and *2* genes, which are linked to oxidative stress and immune responses [16, 17]. DNA methylation of cg17086398 in *SERINC2* was inversely associated with myristoylated alanine-rich C-kinase substrate like 1 (*MARCKSL1*) expression, which is involved in migration of cancer cells [18]. DNA methylation at cg20045320 in *IFITM3* was associated with lower expression of *IFITM3* and *IRF*, which have a critical role in immune responses (Supplementary Figure 3B and Supplementary Table 20) [6, 19].

We finally used functional mapping and annotation to examine tissue-specific expression. Genes identified in the fully-adjusted model showed universal expression at varying levels across tissues. *IFITM3* was highly expressed in all tissues; *BMPR1B* showed low expression across all tissues, except for moderate expression in the prostate and tibial nerve. Remaining genes had moderate or low expression in a wide range of tissues, except for *SERINC2,* which showed high expression in the liver, kidney, salivary gland, and esophagus. *MIR1973* was not represented in the dataset (Figure 3D).

### Mendelian randomization

To evaluate the causal relationship of FDR-significant CpGs to mortality-related risk factors and diseases, we included two sets of Mendelian randomization analysis using methQTL data from KORA and publicly available ARIES data. Only two FDR-significant CpGs (cg18424841 and cg09615688) overlapped with methQTLs in either KORA or ARIES and with SNPs associated with coronary heart disease (CHD) or kidney function. A GWAS assessing longevity and age-related chronic diseases (CHD and kidney function) [34–38] showed no overlap with KORA and ARIES methQTLs even when using a moderate threshold for proxy variants (proxy r^2^ > 0.75). In KORA, cg09615688 showed evidence of a positive causal effect on CHD (OR = 1.51; 95% CI = 1.02, 2.23; Wald ratio method), directionally consistent with the association of overall meta-analysis on mortality. However, this causal estimate at this site was not represented in ARIES methQTL data. Cg18424841 had multiple variants in KORA methQTL data and a single variant in ARIES methQTL data. We did not observe consistent evidence of a causal effect of cg18424841 on CHD. Indeed, weak evidence for a causal effect of cg18424841 on CHD was observed in ARIES using the Wald ratio method but not in KORA using pleiotropy-robust, multi-variant, or Wald ratio methods. We did not find evidence for a causal effect of cg18424841 on kidney function in either KORA or ARIES (Supplementary Table 21).

### Cell-type fractions and all-cause mortality

Cell-type fractions, mostly neutrophil–lymphocyte ratio (NLR), have been often associated with comorbidities and mortality and have been recognized to influence DNA methylation levels [20–22]. We identified that NLR was significantly associated with all-cause mortality only when data were not adjusted for Houseman cell proportions using NAS data (Supplementary Table 22). Interestingly, NLR had no significant association with all-cause mortality when we adjusted for DNA methylation levels at cg07839457, mapped to immune-related gene *NLRC5*. However, the contribution of NLR on mortality at that specific site may be minimized due to adjustment of prior history of cancer and comorbidities in all models.

## DISCUSSION

This study is the largest to date investigating site-specific DNA methylation and all-cause mortality. We identified new whole blood DNA methylation marks that predict all-cause mortality risk, independent from chronological age, lifestyle habits, and morbidity. These newly identified sites may be useful in developing clinical tools for risk assessment and mortality preventive intervention strategies.

All nine FDR-significant CpGs demonstrated novel association with all-cause mortality and were not part of epigenetic aging clocks or mortality risk scores [9, 11–13]. Further, the CpGs were associated with mortality independent from epigenetic aging and mortality signatures. All-cause mortality was associated with a mortality risk score in a model including seven FDR-significant CpGs, although those associations may be driven by the inclusion of CpGs related to our FDR-significant sites. This suggests that whole blood DNA methylation levels at FDR-significant CpGs may be sentinels for epigenetic disruptions leading to aging acceleration and contributing to mortality. In addition, the association between DNA methylation levels at FDR-significant CpGs with chronological aging may suggest that those CpGs are stronger independent biomarkers of aging than other epigenetic aging signatures.

In previous CHARGE meta-analyses [3, 4], DNA methylation of two of the newly-identified CpGs, cg20045320 and cg07839457 (mapping to interferon induced transmembrane protein 3 [*IFITM3*] and *NLRC5*) were respectively associated with smoking and cardiovascular-related chronic inflammation, both factors of mortality. Cardiovascular disease, especially CHD, is a major contributor to mortality [23]. The direction of association with incident heart disease was consistent with that of all-cause mortality. Thus, DNA methylation at these CpGs may contribute to development and progression of CHD and, consequently, to risk of death. To validate this idea, we used a Mendelian randomization approach and identified one site, cg09615688, with a causal effect on CHD in KORA data and weak evidence for the causal effect of cg18424841 on CHD in ARIES data.

Expression of several genes mapped to the fully-adjusted FDR-significant CpGs has been associated with mortality predictors and mortality. Elevated and persistent gene expression levels of *NLRC5*, a master regulator of the immune response [16], has demonstrated an inverse correlation with familial longevity and mortality predictors, such as elevated blood pressure, arterial stiffness, chronic levels of inflammatory cytokines, metabolic dysfunction, and oxidative stress [5]. In addition, expression of *IFITM3* provides an essential barrier to influenza A virus infection *in vivo* and *in vitro*. Absence of *IFITM3* leads to uncontrolled viral replication and a predisposition to morbidity and subsequent mortality [6]. Further, expression of *BMPR1B* enhances cancer cell migration, and approaches targeting *BMPR1B* inhibit metastatic activity in breast cancer [7]. Finally, expression of *MIR1973*, part of a family of microRNAs, increases resistant lung adenocarcinoma cells, with subsequent low apoptosis intensity [8]. This body of evidence may suggest an active role of DNA methylation levels in regulating relevant gene expression and reducing all-cause mortality risk.

The overall meta-analyses included 12 cohorts with varying biological age and mortality. There was a balance between six studies with long (≥10 years) and six cohorts with short (<10 years) average time to follow-up or death. All cohorts showed consistency in magnitudes and directionality for the association with mortality of four CpGs (cg12619262, cg20045320, cg07839457, cg18424841). Two studies (FHS study 1 and KORA) showed non-significant opposing directionality when compared with the rest of the cohorts for several CpGs (FHS-Study 1: cg14866069, cg23666362, cg09615688; KORA: cg17086398, cg14866069, cg23666362). However, both cohorts had among the shortest average time-to-death (FHS-Study 1: 6.1 years; KORA: 4.4 years) and youngest average population age (FHS-Study 1: 65 years; KORA: 61 years). Both cohorts also had limited contribution in our meta-analysis due to reduced number of deaths (FHS-Study 1: 62; KORA: 42). Our results may indicate that DNA methylation levels at these select CpGs were relevant for mortality risk prediction of longer time-to-death in both adults and older-age adults.

Cell-type fractions, including NLR, as related to cancer and systemic inflammation have been related to mortality in different populations [20–22]. When we excluded Houseman cell proportions, NLR was strongly associated with mortality at all CpGs except cg07839457, which is mapped to the immune-related gene *NLRC5*. This may suggest that the contribution of NLR on mortality is minimized when controlled for prior history of cancer and related comorbidities.

In summary, we identified nine CpGs with a novel association with all-cause mortality, responsive to several external stimuli including alcohol consumption and smoking, and more than 10 years before death. These sites thus may be considered sentinels for epigenetic disruptions leading to age-related disease, such as cardiovascular disease, and contributing to mortality. Further studies have to confirm these associations in other tissues and in different populations.

## MATERIALS AND METHODS

### Participating cohort studies

Our meta-analysis included 12,300 participants from 12 population-based cohorts of the Heart and Aging Research in Genetic Epidemiology Consortium (CHARGE; Supplementary material): Atherosclerosis Risk In Communities (ARIC), two studies from the Framingham Heart Study (FHS), Invecchiare in Chianti (InChianti), Kooperative Gesundheitsforschung in der Region Augsburg (KORA), Lothian Birth Cohort 1921 (LBC1921) and 1936 (LBC1936), Normative Aging Study (NAS), UK Adult Twin Registry (TwinsUK), and three studies from the Women’s Health Initiative (WHI), including Broad Agency Announcement 23 (WHI-BAA23) and Epigenetic Mechanisms of PM-Mediated CVD Risk (WHI-EMPC), both European (WHI-EMPC-EA) and African American ancestries (WHI-EMPC-AA). For each participant, we derived years of follow-up using time between the blood draw used for DNA methylation analysis and death or last follow-up. Each cohort excluded participants with diagnosed leukemia (ICD-9: 203–208) or undergoing chemotherapy treatment, which both modify blood-derived data [24, 25]. All participating cohorts shared cohort descriptive statistics and results files from pre-specified in-house mortality analyses (Figure 1). Further information about death ascertainment, covariates measurement and harmonization, protocols, and methods of each cohort are included in the Supplemental Materials. The institutional review committees of each cohort approved this study, and all participants provided written informed consent. Data and analytical codes that support our findings are available from the corresponding author upon request.

### Blood DNA methylation measurements and quality control

Each cohort independently conducted laboratory DNA methylation measurements and internal quality control. All samples underwent bisulfite conversion via the EZ-96 DNA Methylation kit (Zymo Research) and were processed with the Illumina Infinium HumanMethylation450 (450K) BeadChip (Illumina) at Illumina or in cohort-specific laboratories. Quality control of samples included exclusion on the basis of Illumina’s detection *P*-value, low sample DNA concentration, sample call rate, CpG specific percentage of missing values, bisulfite conversion efficiency, gender verification with multidimensional scaling plots, and other quality control metrics specific to cohorts. Each cohort used validated statistical methods for normalizing methylation data on untransformed methylation beta values (ranging 0–1). Some cohorts also made independent probe exclusions. Further details are provided in the Supplemental Material. For meta-analysis, additional probe exclusions were made across all cohorts. In detail, we also excluded control probes, non-CpG sites, probes that mapped to allosomal chromosomes, cross-reactive CpGs, probes with underlying SNPs within 10 bp of the CpG sequence, non-varying CpGs defined by interquartile range of <0.1%, CpGs with ≥10% of missing information, and CpGs with non-converging results [26–28]. We included only CpGs that were available in more than three cohorts. A total of 426, 724 CpGs were included in the meta-analysis (Supplementary Table 5).

The official gene name of each CpG site was noted via Illumina’s genome coordinate. We used the name provided by Illumina with the UCSC Genome Browser and annotation data in Bioconductor. All annotations use the human February 2009 (GRCh37/hg19) assembly.

### Cohort-specific statistical analyses

Each cohort independently ran a common pre-specified statistical analysis in R.version 3.5.1. We estimated the association between locus-by-locus blood DNA methylation levels and all-cause mortality in each cohort using a Cox-regression model. Proportional hazard assumptions were confirmed for each model in all cohorts. Familial relationship was also accounted for, when appropriate, in the model; FHS analyses included cluster for family structure, and TwinsUK analyses used random intercepts for zygosity and family structure. To avoid non-convergent results, cohorts with low deaths (KORA and TwinsUK) used a two-step analysis, in which covariates were first linearly regressed on each probe, and then residuals were used to perform a Cox mortality analysis.

Each cohort adjusted for harmonized covariates in the basic model: age (categories for decades), sex, and technical covariates (plate, chip, row, and column). A second set of fully-adjusted analyses adjusted for this initial list of covariates in addition to education level, self-reported recreational physical activity, smoking status, cumulative smoking (pack-years), body mass index, alcohol intake, hypertension, diabetes, and any personal history of cancer. Cohorts independently estimated cell type proportions using the reference-based Houseman method, which was subsequently extended by Horvath. Cell type correction was applied by including estimated cell type proportions (CD4T, NK cells, monocytes, granulocytes, plasma B cells, CD8T naïve, and memory and effector T cells) as covariates in cohort-specific statistical models. Each cohort underwent statistical validation of Cox-proportional hazard assumptions before being included in the meta-analysis.

### Meta-analysis

We performed inverse variance-weighted fixed-effects meta-analysis. Due to the variability of available CpG sites across cohorts after quality-control steps, we included only CpG sites that were available in three or more cohorts. We accounted for multiple testing by controlling at 5% both the Bonferroni correction and false discovery rate (FDR) using the Benjamini-Hochberg procedure.

For FDR-significant CpGs, we confirmed robustness of the models and results in additional analyses using the leave-one-out cohort validation method, by excluding one cohort at a time and then comparing model estimates for each CpG. We compared effect hazard ratio (HR) and 95% confidence interval (95% CI) for the model to estimates for our models to evaluate the consistency of our findings. For each CpG, we evaluated goodness of the meta-analysis model using the I² statistic measure of inter-study variability from random-effect meta-analyses.

### Enrichment analysis

We enriched our results using a publicly available catalog of all published GWAS relating genetic variants with human diseases (National Human Genome Research Institute-EBI GWAS Catalog) to elucidate potential associations [14]. Enrichment analysis was performed in R using one-sided Fisher exact test. We controlled for false positives with the FDR procedure.

We evaluated whether CpG sites associated with mortality were enriched with genomic features provided in the Illumina annotation file (version 1.2; http://support.illumina.com/array/array_kits/infinium_humanmethylation450_beadchip_kit/downloads.html) to identify CpG location relative to the gene (i.e., body, first exon, 3’-UTR, 5’-UTR, within 200 bp of transcriptional start site [TSS200]), and within 1500 bp of transcriptional start site [TSS1500]) and relation of the CpG site to a CpG island, northern shelf, northern shore, southern shelf, and southern shore.

We also tested each gene mapped to the newly identified CpGs for tissue-specific expression using data from the Genotype Tissue Expression (GTEx) project as integrated by the Functional Mapping and Annotation (FUMA) tool [29], which allowed us to extract and interpret relevant biological information from publicly available repositories and provide interactive figures for prioritized genes. As a result, we obtained a heatmap of genes with normalized gene expression values (reads per kilo base per million). To obtain differentially expressed gene sets for each of 53 tissue types in the database, we used two-sided Student’s *t*-tests on normalized expression per gene per tissue against all other tissues. We controlled for multiple comparison with Bonferroni correction. Finally, we distinguished between genes upregulated and downregulated in a specific tissue compared to other tissues by accounting for sign of the *t*-score [29].

### Pathway analyses

To functionally interpret the genomic information identified from FDR-significant CpGs, we used the Kyoto Encyclopedia of Genes and Genomes (KEGG) pathway database, which links genomic information with higher-order functional information. Genomic information stored in the GENES database is a collection of gene catalogs for all completely sequenced genomes and some partial genomes with up-to-date annotation of gene functions. Higher-order functional information stored in the PATHWAY database contains graphical representations of cellular processes, such as metabolism, membrane transport, signal transduction, and cell cycle [30]. We controlled our results for multiple comparisons with the FDR approach. We finally confirmed our results with the Database for Annotation, Visualization and Integrated Discovery (DAVID). We tested for enrichment in gene ontology biological processes and applied the Benjamini-Hochberg procedure to control for false positivity. We mapped each CpG significantly associated with mortality to genes on the basis of the 450K BeadChip annotation file. We excluded CpGs lacking annotated genes within 10 Mb (n = 3). Using topGO in R, we tested for gene enrichment over the background array (16, 119 unique annotated Entrez Gene IDs) by using Fisher’s exact tests with a minimum of two genes per node.

### Integrating DNA methylation with quantitative trait loci analysis (meQTL)

A subset of 713 KORA samples was genotyped on an Affymetrix Axiom array. We removed variants with a call rate of <0.98, Hardy-Weinberg equilibrium *P* < 5x10^-6^, and minor allele frequency < 0.01. We considered only variants with an information score > 3. Imputation was performed using the 1000 Genomes Project phase I version 3 reference panel with IMPUTE 2.3.0. Phasing of data was performed using SHAPEIT v2. We retained approximately 10,000,000 variants for analyses. In each model, we used DNA methylation beta values as independent variables and SNPs as dependent variables. We adjusted each model for age, sex, body mass index, and white blood cell proportions. We used OmicABEL [31] for the analyses and genotype probabilities for each variant. Due to large size of the output, we retained only variants with *P* < 1x10^-4^. We considered genome-wide significant results at *P* < 1x10^-14^. We reported only associations with CpGs significant in the epigenome-wide association study.

### Integrating DNA methylation with gene expression (eQTM)

In KORA, 998 individuals had both valid methylation and blood gene expression data, which we used to assess whether DNA methylation was correlated with gene expression. Gene expression data (Illumina HumanHT-12 v3 Expression BeadChip) was quality controlled with GenomeStudio, and samples with <6,000 detected genes were excluded from analysis. All samples were log2-transformed and quantile-normalized using the Bioconductor package lumi [32]. A total of 48,803 expression probes passed quality control. We used R (version 3.3.1) to run a linear mixed effects model adjusting for covariates (age, sex, blood cell proportions, and technical variables of RNA integrity number, sample storage time, and RNA amplification batch) and a random intercept for RNA amplification batch. Models were run for each of the nine newly-identified CpGs associated with mortality. We filtered results to report only CpG-expression probe pairs located on the same chromosome. Start and end sites for each gene were determined according to the Illumina HT annotation file. A cutoff of 500,000 bp was used to differentiate cis- vs. trans-eQTMs.

### Miettinen’s population attributable factor and mendelian randomization analysis

To assess the contribution of methylation levels of each CpG to all-cause mortality, we calculated Miettinen’s population attributable fraction on data from the in-house Normative Aging Study (NAS) and Women Health Initiative-Epigenetic Mechanisms of Particulate Matter-Mediated Cardiovascular Disease (WHI-EMPC) for European and African American ancestries. Population attributable fraction takes into account strength of association between the risk factor (DNA methylation higher than the mean in specific CpG sites) and outcome (mortality) as well as prevalence of the risk factor in the population [33]. This metric provides estimates of the public health importance of risk factors, ascertaining what proportion of the outcome is due to exposure to the risk factor, and distinguishes between etiologic fraction attributable to or related to the given risk factor depending on whether all or just some confounding by extraneous factors was under control [33]. To support information about the population attributable factor, we also included two Mendelian randomization approaches.

We identified the causal effect on all-cause mortality of FDR-significant CpGs by using two sample Mendelian randomization analyses and summary statistics from published GWAS for chronic diseases and longevity [34] and chronic diseases, including CHD [35], kidney function (serum creatinine), [36] blood pressure, [37] and type 2 diabetes [38]. We extracted GWAS information with MR-base [14]. We also extracted SNP-methylation association summary statistics from both KORA and publicly available ARIES [39] methQTL data; for ARIES, we used MR-base [40]. To account for multiple variants and pleiotropy, we used multiple Mendelian randomization methods—when only one variant was present, we used the Wald Ratio method [41]; when we had multiple variants, we used MR Egger [42], weighted median [43], and weighted mode [44], as these three methods use different assumptions to provide consistent causal effect estimates even with invalid instruments arising from horizontal pleiotropy, a primary source of bias in multi-variant Mendelian randomization analyses.

### FDR-significant CpGs, DNA methylation-related aging measures, and mortality risk score

PhenoAge, a composite measure of CpG sites representing phenotypic age, captures differences between lifespan and health span. The Horvath clock is a linear combination of sites identifying the cumulative effect of an epigenetic maintenance system [1, 45]. Among the 513 CpGs comprising PhenoAge, 41 are shared with the Horvath clock. While both aging measures correlate strongly with age in every tissue and cell type tested, and both captured risks for mortality across multiple tissues and cells, PhenoAge is highly predictive of nearly every morbidity [1, 10]. Blood PhenoAge outperformed the Horvath clock with regard to predictions for a variety of aging outcomes, including all-cause mortality. The mortality risk score instead was based on results using discovery cohort ESTHER (61 years old on average) and both ESTHER and KORA for validation [11].

To investigate whether the association of FDR-significant CpGs with mortality was independent of DNA methylation aging measures and risk score, we included acceleration of PhenoAge and Horvath clock, defined respectively as discrepancies between age with PhenoAge and Horvath clock age and the risk score. We also identified the correlation between each CpG included in the risk score and our FDR-significant CpGs, and we compared our pooled meta-analysis results with previous findings.

### Cell-type fractions and all-cause mortality

Cell-type fractions, mostly NLR, influence DNA methylation levels and have been associated with comorbidities and mortality [20–22]. To elucidate which cell proportions were associated with mortality when adjusting for DNA methylation at FDR-significant CpGs, we included NLR, which has been associated with lung cancer risk and mortality [21] as well was cardiovascular disease and mortality in prospective studies [22]. NLR computation was performed using DNA methylation data via Koestler et al. [46]

## Supplementary Material

Supplementary Methods

Supplementary Figures

Supplementary Table 1

Supplementary Table 2

Supplementary Tables 3-5, 7-9, 11, 13-16, 18-22

Supplementary Table 6

Supplementary Table 10

Supplementary Table 12

Supplementary Table 17
