# Supplementary material for: Blood DNA methylation sites predict death risk in a longitudinal study of 12,300 individuals
Source: Aging (Albany NY). 2020 Jul 22;12(14):14092–124. doi: 10.18632/aging.103408 (PMC7425458; doi:10.18632/aging.103408)
Supplement: Supplementary Methods [file aging-12-103408-s007..pdf]

SUPPLEMENTARY METHODS

Supplementary material - cohort description

The atherosclerosis risk in communities (ARIC) study

ARIC cohort description

The ARIC Study is a population-based prospective cohort study of cardiovascular disease risk in four US communities [1]. Between 1987 and 1989, 7,082 men and 8,710 women aged 45–64 years were enrolled in Forsyth County, North Carolina; Jackson, Mississippi (African Americans only); suburban Minneapolis, Minnesota; and Washington County, Maryland. The ARIC Study protocol was approved by the institutional review board of each participating university, and participants provided written informed consent. Participants underwent a baseline clinical examination (Visit 1) and four subsequent follow-up clinical exams (Visits 2–5). The present analysis is restricted to African Americans from Jackson and Forsyth County centers. Baseline for mortality follow-up is either Visit 2 (1990–1992) or Visit 3 (1993–1995), when the DNA used for methylation quantification was collected. Covariates were measured at the time of blood draw, unless otherwise specified. Data on education, smoking status, smoking pack-years, alcohol intake, and physical activity were obtained by self-report at Visit 1. Trained technicians took fasting blood samples and measured height and weight using standard protocols. Diabetes was defined as a fasting blood glucose level of ≥126 mg/dL, non-fasting blood glucose level of ≥200 mg/dL, self-reported physician diagnosis of diabetes, or use of antidiabetic medication in the past 2 weeks. Hypertension was defined as systolic blood pressure ≥140 mm Hg, diastolic blood pressure ≥90 mm Hg, or self-reported use of antihypertensive medication in the past 2 weeks. History of cancer was defined by self-report or incident cancer cases found between Visit 1 and time of blood draw found through cancer registry and hospital linkage. History of coronary heart disease (CHD) was defined as self-reported history at baseline or an adjudicated event (Myocardial infarction (MI), silent MI, coronary artery bypass surgery, or angioplasty) found between Visit 1 and time of blood draw.

ARIC death ascertainment

Deaths among cohort participants were identified through December 2012 via annual telephone calls and by surveillance of local death certificates and obituaries. If a participant was lost to telephone follow-up, a National Death Index search was conducted.

ARIC DNA methylation quantification

Genomic DNA was extracted from peripheral blood leukocyte samples using the Gentra Puregene Blood Kit (Qiagen; Valencia, CA, USA) according to the manufacturer's instructions (https://www.qiagen.com). Bisulfite conversion of 1 µg genomic DNA was performed using the EZ-96 DNA Methylation Kit (Deep Well Format) (Zymo Research; Irvine, CA, USA) according to the manufacturer's instructions (https://www.zymoresearch.com). Bisulfite conversion efficiency was determined by PCR amplification of converted DNA before proceeding with methylation analyses on the Illumina platform using Zymo Research's Universal Methylated Human DNA Standard and Control Primers. The Illumina Infinium HumanMethylation450K Beadchip array (HM450K) was used to measure DNA methylation (Illumina, Inc.; San Diego, CA, USA). Background subtraction was conducted with the GenomeStudio software using built-in negative control bead types on the array. Positive and negative controls and sample replicates were included on each 96-well plate assayed. After exclusion of controls, replicates, and samples with integrity issues or failed bisulfite conversion, a total of 2,841 study participants had HM450K data available for further quality control (QC) analyses. We removed poor-quality samples with pass rate of <99% (i.e., if the sample had at least 1% of CpG sites with detection P-value > 0.01 or missing), indicative of lower DNA quality or incomplete bisulfite conversion, and samples with a possible gender mismatch based on evaluation of selected CpG sites on the Y chromosome. Additional details have been published elsewhere [2 , 3].

Framingham heart study offspring cohort (FHS)

FHS study participants

The FHS Offspring Cohort began enrollment in 1971 and included 5,124 offspring of the FHS original cohort as well as spouses of the offspring. Participants were eligible for the current study if they attended the eighth examination cycle (2005–2008) and consented to have their DNA used for genetic research. All participants provided written informed consent at the time of each examination visit. The study protocol was approved by the Institutional Review Board at Boston University Medical Center (Boston, MA). FHS data are available in dbGaP (accession number: phs000724.v2.p9).

FHS death ascertainment

Deaths among FHS participants that occurred before January 1, 2013 were ascertained using multiple strategies, including routine contact with participants for health history updates, surveillance at the local

hospital, obituaries in the local newspaper, and queries to the National Death Index. Death certificates, hospital and nursing home records before death, and autopsy reports were requested. When cause of death was undeterminable, the next of kin were interviewed. The date and cause of death were reviewed by an endpoint panel of three investigators.

FHS DNA methylation quantification

Peripheral blood samples were collected at the 8th examination. Genomic DNA was extracted from buffy coats using the Gentra Puregene DNA extraction kit (Qiagen) and bisulfite converted using the EZ DNA Methylation kit (Zymo Research). DNA methylation quantification was conducted in two laboratory batches using the Illumina Infinium HumanMethylation450 array. Methylation beta values were generated using the Bioconductor *minfi* package with background correction. Sample exclusion criteria included poor SNP matching of control positions, missing rate >1%, outliers from multi-dimensional scaling, and sex mismatch. In addition, we excluded individuals with leukemia and those who received chemotherapy. Additional sample exclusions included those with mismatches in their reported sex and methylation-predicted sex as well as methylation-predicted tissues that were not blood. Lastly, samples with correlation with our reference population of r < 0.80 were excluded. Predicted sex, tissues, correlation with reference population, and DNA methylation-predicted ages were computed using our online age calculator (http://labs.genetics.ucla.edu/horvath/dnamage). Background subtraction was applied using the *preprocessIllumina* command in the *minfi* Bioconductor package [4]. In total, 2,635 samples and 443,304 CpG probes remained for analysis.

Invecchiare in chianti (InCHIANTI) study

InChianti study participants

The InCHIANTI Study is a population-based prospective cohort study of residents ≥20 years old from two areas in the Chianti region of Tuscany, Italy. Sampling and data collection procedures have been described elsewhere [5]. Briefly, 1,326 participants donated a blood sample at baseline (1998–2000), of which 784 also donated a blood sample at 9-year follow-up (2007–2009). DNA methylation was assayed using the Illumina Infinium HumanMethylation450 platform in DNA samples corresponding to participants with sufficient DNA at both baseline and Year 9 visits (n = 499). All participants provided written informed consent to participate in this study. The study complied with the Declaration of Helsinki. The Italian National Institute of Research and Care on Aging Institutional Review Board approved the study protocol.

InChianti death ascertainment

Vital status was ascertained using data from the Tuscany Regional Mortality General Registry. Deaths were assessed until December 1, 2014.

InChianti DNA methylation quantification

Genomic DNA was extracted from buffy coat samples using an AutoGen Flex and quantified on a Nanodrop1000 spectrophotometer before bisulfite conversion. Genomic DNA was bisulfite converted using the Zymo EZ-96 DNA Methylation Kit (Zymo Research) per the manufacturer's protocol. CpG methylation status of 485, 577 CpG sites was determined using the Illumina Infinium HumanMethylation450 BeadChip per the manufacturer's protocol, as previously described [6]. Initial data analysis was performed using GenomeStudio 2011.1 (Model M Version 1.9.0, Illumina Inc.). Threshold call rate for inclusion of samples was 95%. Quality control of sample handling included comparison of clinically reported sex versus sex of the same samples determined by analysis of methylation levels of CpG sites on the X chromosome [6]. Background subtraction was applied using the *preprocessIllumina* command in the *minfi* Bioconductor package [4].

Cooperative health research in the region of augsburg (KORA) F4 cohort

KORA cohort description

The KORA study is an independent population-based cohort from Augsburg, Southern Germany. Whole blood samples of the KORA F4 survey (examination 2006–2008), a seven-year follow-up study of the KORA S4 cohort, were used. Out of 4,621 participants for the KORA S4 baseline study, 3,080 participants participated in the KORA F4 follow-up study [7]. Participants provided written informed consent, and the study was approved by the local ethics committee (Bayerische Landesärztekammer). For 1,799 subjects, methylation data as well as information about death ascertainment was available. Before analyses, all individuals with a detection *P*-value > 0.05 for >1% of probes were removed (375 individuals). Sex checks performed during calculation of DNAmAge resulted in the removal of another 167 individuals, 137 of whom had an "unsure" gender. This left 1,257 individuals for analysis. At the KORA F4 follow-up examination, all individuals completed questionnaires and physical examinations conducted by trained staff covering demographics, lifestyle, and medical history since the KORA S4 examination. Collected information included age, sex, years of education, smoking status (current regular, current irregular, former, never), pack-years, alcohol consumption

(g/day), physical activity (active, inactive), diabetes status, hypertension status, self-reported cancer diagnosis, and body mass index (BMI), among other clinical variables [7].

KORA mortality ascertainment

The vital status of all F4 participants was ascertained through the population registries inside and outside the study area in 2011 (cut-off date: December 31, 2011). Record linkage was based on name, sex, date of birth, and address. If the person died, the time and location of death was assessed via population registries, and a copy of the death certificate was obtained from the Regional Health Department. If the person moved out of the study area, time of the move and information on the new address was typically available. Vital status could not be assessed for those who had moved to a foreign country or to an unknown location in the country. Causes of death were ICD-9 revision coded. There were a total of 42 deaths, including 16 from cardiovascular disease and 17 from cancer.

KORA DNA methylation measures

Whole blood was drawn into serum gel tubes. We bisulfite-converted 1 μg of genomic DNA using the EZ-96 DNA Methylation Kit (Zymo Research) according to the manufacturer's procedure, with the alternative incubation conditions recommended when using the Illumina Infinium Methylation Assay. Genome-wide DNA methylation was analyzed in 1,799 subjects using the Illumina Infinium HumanMethylation450 BeadChip Array. Raw methylation data were extracted using the Illumina Genome Studio (version 2011.1) with the methylation module (version 1.9.0). Preprocessing was performed with R (version 3.0.1). Probes with signals from less than three functional beads and probes with a detection *P*-value > 0.01 were defined as low-confidence probes. Probes that covered SNPs (MAF in Europeans > 5%) were excluded from the data set. A color bias adjustment was performed with the R package lumi (version 2.12.0) by smooth quantile normalization and background correction based on negative control probes present on the Infinium HumanMethylation BeadChip. This was performed separately for the two-color channels and chips. β-values corresponding to low-confidence probes were set to missing. A 95% call rate threshold was applied on samples and CpG sites. Beta-mixture quantile normalization (BMIQ) was applied by using the R package wateRmelon, version 1.0.3. Plate and batch effects were investigated by principle component analysis and eigenR2 analysis, because KORA F4 samples were processed on 20 96-well plates across nine different batches.

Probes with a detection $P > 0.05$ for > 1% of samples were removed as well as all "ch" and "rs" probes, leaving a total of 431, 217 probes for analysis. Although raw beta values were used in Dr. Horvath's online calculator to determine cell counts, normalized data was used for the final analyses.

To reduce non-biological variability between observations, data were normalized using quantile normalization on raw signal intensities. Precisely, quantile normalization was stratified to six probe categories based on probe type and color channel (i.e., Infinium I signals from beads targeting methylated CpG sites obtained through red and green color channels, Infinium I signals from beads targeting unmethylated CpG sites obtained through red and green color channels, and Infinium II signals obtained through red and green color channels [8]) using the R package *limma*, version 3.16.5 [9]. Further, to correct the shift in the distribution of methylation values observed for the two different assay designs (Infinium I and Infinium II) on the BeadChip, BMIQ was applied [10] using the R package wateRmelon, version 1.0.3 [11].

Lothian birth cohorts of 1921 and 1936 (LBC1921 and LBC1936)

LBC cohort description

LBC1921 and LBC1936 are two longitudinal studies of aging [12, 13] that derive from the Scottish Mental Surveys of 1932 and 1947, respectively, when nearly all 11-year-old children in Scotland completed a test of general cognitive ability [14]. Survivors living in the Lothian area of Scotland were recruited in late-life at a mean age of 79 years for LBC1921 (n = 550) and mean age of 70 years for LBC1936 (n = 1,091). Follow-up took place at ages 70, 73, and 76 years in LBC1936 and ages 79, 83, 87, and 90 years in LBC1921. Collected data include genetic information, longitudinal epigenetic information, longitudinal brain imaging (LBC1936), numerous blood biomarkers, and anthropomorphic and lifestyle measures. Post-QC, DNA methylation data were available for 920 LBC1936 participants at age 70 years and for 446 LBC1921 participants at age 79 years. At each in-person visit, participants completed questionnaires regarding demography, lifestyle, and medical history. They reported chronological age, years of education, smoking status (never, former, current), pack-years consumption (continuous), alcohol consumption (light, moderate, and heavy drinkers), self-reported type 2 diabetes, cancer, and hypertension. BMI was computed from anthropometric measures. Participants were asked to remove their shoes before a SECA stadiometer was used to assess height in centimeters. Weight (after removing shoes and outer clothing) was measured in

kilograms using a digital readout from electronic SECA scales.

LBC mortality ascertainment

For both LBC1921 and LBC1936, mortality status was obtained via data linkage from the National Health Service Central Register, provided by the General Register Office for Scotland (now National Records of Scotland). Participant deaths and cause of death are routinely flagged to the research team about every 12 weeks. The last update available for the current project was 26th November 2014.

LBC DNA methylation measures

Detailed information about collection and QC steps on LBC methylation data have been reported previously [12, 15]. Briefly, the Illumina Infinium HumanMethylation450 BeadChip was used to measure DNA methylation in whole blood of consenting participants. Background correction was performed, and QC was used to remove probes with a low detection rate, low quality (manual inspection), and low call rate as well as samples with a poor match between genotypes and SNP control probes or incorrect predicted sex. Additional QC was performed to remove samples and probes in which >1% of probes or samples, respectively, had a detection $P > 0.05$. The working set included 442,227 CpG probes.

Normative aging study (NAS)

NAS cohort description

The ongoing longitudinal US Department of Veterans Affairs NAS was established in 1963 and included men 21–80 years old and free of known chronic medical conditions at entry [16]. Participants were invited to medical examinations every three to five years. At each visit, men provided information on medical history, lifestyle, and demographic factors and underwent physical examinations and laboratory tests. DNA samples were collected from 675 active participants between 1999–2007 [16]. We excluded participants who were non-white or who reported leukemia at the time of DNA extraction, leaving a total of 646 individuals with a single observation each. Participants provided written informed consent at each visit. The NAS study was approved by the institutional review boards of participating institutions. At each in-person visit, participants completed questionnaires regarding demography, lifestyle, and medical history. They reported chronological age, years of education, smoking status (never, former, current), pack-years consumption (continuous), alcohol consumption (<2, ≥2 drinks/day), physical activity (<12, 12–30, ≥30 metabolic equivalent hours [MET-h] per week), type 2 diabetes (self-reported diagnosis and/or use of diabetes medications), diagnosis of CHD (validated on medical records, ECG, and physician exams), diagnosis of malignant cancer in the five years prior the visit (diagnosed with ICD-9 code). High blood pressure was defined as antihypertensive medication use, systolic blood pressure ≥140 mmHg, or diastolic blood pressure ≥90 mmHg at study visit. BMI was computed from anthropometric measures, performed with participants in undershorts and socks [17].

NAS mortality ascertainment

Official death certificates were obtained for decedents from the appropriate state health departments and were reviewed by a physician. An experienced research nurse coded the cause of death using ICD-9. Both participant deaths and causes of death were routinely updated by the research team, and the last update available was December 31, 2013 [12].

NAS DNA methylation measures

DNA was extracted from buffy coats using the QIAamp DNA Blood Kit (Qiagen). We used 500 ng of DNA for bisulfite conversion using the EZ-96 DNA Methylation Kit (Zymo Research). To reduce chip and plate effects, we used a two-stage age-stratified algorithm to randomize samples and ensure similar age distributions across chips and plates; 12 samples that were sampled across all age quartiles were randomized to each chip, and then chips were randomized to plates (8 chips/plate).

QC analysis was performed to remove samples and probes, where >1% of probes or samples, respectively, had a detection $P > 0.05$. Remaining samples were preprocessed using the Illumina-type background correction [18] and normalized with dye-bias [19] and BMIQ [20] adjustments, which were used to generate beta methylation values. The working set included 477, 928 CpG probes. DNA methylation age was computed using the Horvath calculator from background-corrected methylation data, and QC analysis was performed only on samples, leaving 485, 512 CpG and CpH probes in the working set.

TwinsUK

TwinsUK study participants

The TwinsUK cohort was established in 1992 and recruited both monozygotic and dizygotic same-sex twins in the United Kingdom. The majority of participants are female, Caucasian, and mostly disease-free at time of ascertainment. There are >13, 000 twin participants in the cohort, of which 805 were included in the current study. Whole blood samples were collected during participants' clinical visits, along with questionnaire data on phenotype and lifestyle factors. All subjects provided written informed consent [21].

Information on physical activity, smoking pack-years, plate number, and chip position number were not available for subjects in the TwinsUK dataset and therefore were not adjusted as covariates in all analyses.

TwinsUK death ascertainment

Mortality data were collected using two approaches: 1) during routine contact for standard clinical visits in TwinsUK, and 2) using queries to the National Death Register. Date and cause of death were recorded.

TwinsUK DNA methylation quantification

DNA samples were extracted from whole blood using the DNeasy kit (Qiagen). DNA was bisulfite converted using the EZ DNA methylation kit (Zymo Research). Methylation levels were profiled using the Illumina Infinium HumanMethylation450 array, and methylation betas were generated using the R package *minfi* with background correction. Raw beta levels were subjected to BMIQ dilation to correct for technical effects. Probe exclusion criteria included probes that mapped to multiple locations in the reference sequence and probes in which >1% of subjects had detection $P > 0.05$. Individuals with >5% missing probes, with mismatched sex, and with mismatched genotypes were also excluded. Methylation-predicted sex, methylation-predicted blood cell types, correlations with the reference population, and DNA methylation-predicted age were computed using the online epigenetic age calculator (http://labs.genetics.ucla.edu/horvath/dnamage).

Women's health initiative–broad agency announcement 23 (WHI-BAA23)

WHI-BAA23 cohort description

Subjects included a subsample of participants of the WHI study, a national study that began in 1993 and enrolled postmenopausal women 50–79 years of age into one of three randomized clinical trials. Women were selected from one of two WHI large sub-cohorts that had previously undergone genome-wide genotyping as well as profiling for 7 cardiovascular disease-related biomarkers, including total cholesterol, high-density lipoprotein, low-density lipoprotein, triglycerides, C-reactive protein (CRP), creatinine, insulin, and glucose through two core WHI ancillary studies [22]. The first cohort is the WHI SNP Health Association Resource (SHARe) cohort of minorities that includes >8,000 African American (AA) women and >3,500 Hispanic women. Women were genotyped through the WHI core study M5-SHARe (www.whi.org/researchers/data/WHIStudies/StudySites/M5) and underwent biomarker profiling through WHI Core study W54-SHARe (...data/WHIStudies/StudySites/W54). The second cohort consists of a combination of European Americans (EA) from two hormonal therapy trials selected for GWAS and biomarkers in core studies W58 (.../data /WHIStudies/StudySites/W58) and W63 (.../data/WHIStudies/StudySites/W63). From these two cohorts, two sample sets were formed. Sample Set 1 is a sample set of 637 CHD cases and 631 non-CHD cases as of Sept 30, 2010. Sample Set 2 is a non-overlapping sample of 432 cases of CHD and 472 non-CHD cases as of September 17, 2012. The ethnic groups differed in terms of age distribution, as Caucasian women tended to be older. We acknowledge a potential for selection bias using the above-described sampling scheme in WHI but suspect that if such bias is present, it is minimal. First, selection bias is introduced by restricting our methylation profiling at baseline to women with GWAS and biomarker data from baseline as well, given the requirement that these subjects must have signed the WHI supplemental consent for broad sharing of genetic data in 2005. However, we believe that selection bias at this stage is minimized by inclusion of subjects who died between time of start of the WHI study and time of supplemental consent in 2005, which excluded only ~6%–8% of all WHI participants. Subjects unable or unwilling to sign consent in 2005 may not represent a random subset of all participants who survived to 2005. Second, some selection bias may also occur if similar gross differences exist in the characteristics of participants who consented to be followed in the two WHI extension studies beginning in 2005 and 2010 compared to non-participants at each stage. We believe these selection biases, if present, have minimal effects on our effect estimates. Data are available from this page: https://www.whi.org/researchers/Stories/June%202015%20WHI%20Investigators'%20Datasets%20Released.aspx, as well as https://www.whi.org/researchers/data/Documents/WHI%20Data%20Preparation%20and%20Use.pdf

WHI-BAA23 death ascertainment

We used the variable "DEATHALL" from form 124/120 that incorporated any report of death (as of August 2015).

WHI-BAA23 DNA methylation quantification

In brief, bisulfite conversion using the Zymo EZ DNA Methylation Kit (Zymo Research) as well as subsequent hybridization of the Illumina HumanMethylation450k Bead Chip and scanning (iScan, Illumina) were performed according to the manufacturer's protocols by applying standard settings. DNA methylation levels (β values) were determined by calculating the ratio of intensities between methylated (signal A) and un-methylated (signal B) sites. Specifically, β value was calculated from the intensity of methylated (M corresponding to signal A) and un-methylated (U corresponding to signal B) sites, as the ratio of fluorescent signals $\beta = \text{Max}(M,0)/[\text{Max}(M,0)+\text{Max}$

(U,0)+100]. Thus, β values range from 0 (completely un-methylated) to 1 (completely methylated).

Women's health initiative—epigenetic mechanisms of PM-Mediated CVD risk (WHI-EMPC)

WHI-EMPC cohort description

WHI-EMPC is an ancillary study of epigenetic mechanisms underlying associations between ambient particulate matter (PM) air pollution and cardiovascular disease in the WHI clinical trials (CT) cohort. It is funded by the National Institute of Environmental Health Sciences (R01-ES020836).

The WHI-EMPC study population is a stratified, random sample of 2,200 WHI CT participants who were examined in 1993–2001; had available buffy coats, core analytes, electrocardiograms, and ambient concentrations of PM; and were not taking anti-arrhythmic medications at the time.

As such, WHI-EMPC is representative of the larger, multiethnic WHI CT population from which it was sampled: 68 ,132 participants aged 50–79 years who were randomized to hormone therapy, calcium/vitamin D supplementation, and/or dietary modification in 40 U.S. clinical centers at baseline exam (1993–1998) and re-examined in the fasting state one, three, six, and nine years later [23, 24]. During participant visits, data on age, race/ethnicity, education, smoking status (current, former, never), pack-years of smoking, alcohol consumption (drinks per week), recreational physical activity (MET-hours/week), weight/height/BMI, systolic and diastolic blood pressure, medication use, CHD, type 2 diabetes, and cancer diagnosis were obtained.

Hypertension status was based on systolic blood pressure ≥140 mmHg or diastolic blood pressure ≥90 mmHg or antihypertensive medication use (angiotensin converting enzyme inhibitors, angiotensin II receptor antagonists, beta blockers, calcium channel blockers, thiazides). CHD was defined by a history of myocardial infarction (acute, hospitalized, definite or probable events supported by cardiac pain, electrocardiogram, and biomarker data) or revascularization procedure (coronary artery bypass graft, percutaneous coronary angioplasty, stent) and was self-reported at baseline and confirmed by physician-review, classification, and local/central adjudication of medical records during follow-up. Type 2 diabetes was defined by a self-reported history of physician-treated diabetes, fasting glucose ≥126 mg/dL, non-fasting glucose ≥200 mg/dL, or anti-diabetic medication use. Cancer was defined by a diagnosis of any cancer, excluding leukemia and other hematologic malignancies (Hodgkin's lymphoma, non-Hodgkin's lymphoma, multiple myeloma).

Current analyses involve information collected at the first available visit with available DNA methylation data and stratification by race/ethnicity [European (WHI-EMPC-EA) and African American (WHI-EMPC-AA) ancestries].

WHI-EMPC mortality ascertainment

All-cause mortality and sub-classification of the underlying cause of death to cardiovascular or cancer mortality were based on WHI physician review of death certificates, medical records, and autopsy reports. Cardiovascular disease mortality was defined as death due to definite or possible CHD, cerebrovascular disease, or other or unknown cardiovascular disease. Cancer mortality was defined as death due to any cancer. Participants affected by leukemia or other hematologic malignancies (i.e., Hodgkin's lymphoma, non-Hodgkin's lymphoma, multiple myeloma) were excluded due to known effects on red cell, white cell, and platelet counts.

WHI-EMPC DNA methylation quantification

Genome-wide DNA methylation at CpG sites was measured using the Illumina 450K Infinium Methylation BeadChip, quantitatively represented by beta (percentage of methylated cytosines over the sum of methylated and unmethylated cytosines) and quality-controlled using the following filters: detection $P > 0.01$ in >10% of samples, detection $P > 0.01$ or missing in >1% of probes, and probes with a coefficient of variation <5%, yielding values of beta at 293,171 sites. DNA methylation data were normalized using BMIQ [25] and stage-adjusted using ComBat [10]. Modeled epigenome-wide associations also adjusted for cell subtype proportions (CD8-T, CD4-T, B cell, natural killer, monocyte, and granulocyte) [26] and for technical covariates, including plate, chip, row, and column.

REFERENCES

1. The atherosclerosis risk in communities (ARIC) study: design and objectives. The ARIC investigators. Am J Epidemiol. 1989; 129:687–702. PMID:2646917

2. Demerath EW, Guan W, Grove ML, Aslibekyan S, Mendelson M, Zhou YH, Hedman ÅK, Sandling JK, Li LA, Irvin MR, Zhi D, Deloukas P, Liang L, et al. Epigenome-wide association study (EWAS) of BMI, BMI change and waist circumference in african american adults identifies multiple replicated loci. Hum Mol Genet. 2015; 24:4464–79. https://doi.org/10.1093/hmg/ddv161 PMID:25935004

3. Bose M, Wu C, Pankow JS, Demerath EW, Bressler J, Fornage M, Grove ML, Mosley TH, Hicks C, North K, Kao WH, Zhang Y, Boerwinkle E, Guan W. Evaluation of microarray-based DNA methylation measurement using technical replicates: the atherosclerosis risk in communities (ARIC) study. BMC Bioinformatics. 2014; 15:312. https://doi.org/10.1186/1471-2105-15-312 PMID:25239148

4. Aryee MJ, Jaffe AE, Corrada-Bravo H, Ladd-Acosta C, Feinberg AP, Hansen KD, Irizarry RA. Minfi: a flexible and comprehensive bioconductor package for the analysis of infinium DNA methylation microarrays. Bioinformatics. 2014; 30:1363–69. https://doi.org/10.1093/bioinformatics/btu049 PMID:24478339

5. Ferrucci L, Bandinelli S, Benvenuti E, Di Iorio A, Macchi C, Harris TB, Guralnik JM. Subsystems contributing to the decline in ability to walk: bridging the gap between epidemiology and geriatric practice in the InCHIANTI study. J Am Geriatr Soc. 2000; 48:1618–25. https://doi.org/10.1111/j.1532-5415.2000.tb03873.x PMID:11129752

6. Moore AZ, Hernandez DG, Tanaka T, Pilling LC, Nalls MA, Bandinelli S, Singleton AB, Ferrucci L. Change in epigenome-wide DNA methylation over 9 years and subsequent mortality: results from the InCHIANTI study. J Gerontol A Biol Sci Med Sci. 2016; 71:1029–35. https://doi.org/10.1093/gerona/glv118 PMID:26355017

7. Rückert IM, Heier M, Rathmann W, Baumeister SE, Döring A, Meisinger C. Association between markers of fatty liver disease and impaired glucose regulation in men and women from the general population: the KORA-F4-study. PLoS One. 2011; 6:e22932. https://doi.org/10.1371/journal.pone.0022932 PMID:21850244

8. Bibikova M, Barnes B, Tsan C, Ho V, Klotzle B, Le JM, Delano D, Zhang L, Schroth GP, Gunderson KL, Fan JB, Shen R. High density DNA methylation array with single CpG site resolution. Genomics. 2011; 98:288–95. https://doi.org/10.1016/j.ygeno.2011.07.007 PMID:21839163

9. Smyth GK. Limma: linear models for microarray data. Bioinformatics and computational biology solutions using R and Bioconductor: Springer. 2005; 397–420. https://doi.org/10.1007/0-387-29362-0_23

10. Teschendorff AE, Marabita F, Lechner M, Bartlett T, Tegner J, Gomez-Cabrero D, Beck S. A beta-mixture quantile normalization method for correcting probe design bias in illumina infinium 450 K DNA methylation data. Bioinformatics. 2013; 29:189–96. https://doi.org/10.1093/bioinformatics/bts680 PMID:23175756

11. Pidsley R, Y Wong CC, Volta M, Lunnon K, Mill J, Schalkwyk LC. A data-driven approach to preprocessing illumina 450K methylation array data. BMC Genomics. 2013; 14:293. https://doi.org/10.1186/1471-2164-14-293 PMID:23631413

12. Marioni RE, Shah S, McRae AF, Chen BH, Colicino E, Harris SE, Gibson J, Henders AK, Redmond P, Cox SR, Pattie A, Corley J, Murphy L, et al. DNA methylation age of blood predicts all-cause mortality in later life. Genome Biol. 2015; 16:25. https://doi.org/10.1186/s13059-015-0584-6 PMID:25633388

13. Taylor AM, Pattie A, Deary IJ. Cohort profile update: the lothian birth cohorts of 1921 and 1936. Int J Epidemiol. 2018; 47:1042–42r. https://doi.org/10.1093/ije/dyy022 PMID:29546429

14. Marioni RE, McRae AF, Bressler J, Colicino E, Hannon E, Li S, Prada D, Smith JA, Trevisi L, Tsai PC, Vojinovic D, Simino J, Levy D, et al. Meta-analysis of epigenome-wide association studies of cognitive abilities. Mol Psychiatry. 2018; 23:2133–44. https://doi.org/10.1038/s41380-017-0008-y PMID:29311653

15. Shah S, McRae AF, Marioni RE, Harris SE, Gibson J, Henders AK, Redmond P, Cox SR, Pattie A, Corley J, Murphy L, Martin NG, Montgomery GW, et al. Genetic and environmental exposures constrain epigenetic drift over the human life course. Genome Res. 2014; 24:1725–33. https://doi.org/10.1101/gr.176933.114 PMID:25249537

16. Bell B, Rose CL, Damon A. The veterans administration longitudinal study of healthy aging. Gerontologist. 1966; 6:179–84. https://doi.org/10.1093/geront/6.4.179 PMID:5342911

17. Troisi RJ, Heinold JW, Vokonas PS, Weiss ST. Cigarette smoking, dietary intake, and physical activity: effects on body fat distribution—the normative aging study. Am J Clin Nutr. 1991; 53:1104–11. https://doi.org/10.1093/ajcn/53.5.1104 PMID:1850574

18. Triche TJ Jr, Weisenberger DJ, Van Den Berg D, Laird PW, Siegmund KD. Low-level processing of illumina infinium DNA methylation BeadArrays. Nucleic Acids Res. 2013; 41:e90. https://doi.org/10.1093/nar/gkt090 PMID:23476028

19. Davis S, Du P, Bilke S, Triche T, Jr and Bootwalla M.

methylumi: Handle Illumina methylation R package version 2140 2015.

20. Teschendorff AE, Jones A, Fiegl H, Sargent A, Zhuang JJ, Kitchener HC, Widschwendter M. Epigenetic variability in cells of normal cytology is associated with the risk of future morphological transformation. Genome Med. 2012; 4:24. https://doi.org/10.1186/gm323 PMID:22453031

21. Nica AC, Parts L, Glass D, Nisbet J, Barrett A, Sekowska M, Travers M, Potter S, Grundberg E, Small K, Hedman AK, Bataille V, Tzenova Bell J, et al, and MuTHER Consortium. The architecture of gene regulatory variation across multiple human tissues: the MuTHER study. PLoS Genet. 2011; 7:e1002003. https://doi.org/10.1371/journal.pgen.1002003 PMID:21304890

22. Curb JD, McTiernan A, Heckbert SR, Kooperberg C, Stanford J, Nevitt M, Johnson KC, Proulx-Burns L, Pastore L, Criqui M, Daugherty S, and WHI Morbidity and Mortality Committee. Outcomes ascertainment and adjudication methods in the women's health initiative. Ann Epidemiol. 2003; 13:S122–28. https://doi.org/10.1016/s1047-2797(03)00048-6 PMID:14575944

23. Design of the women's health initiative clinical trial and observational study. The women's health initiative study group. Control Clin Trials. 1998; 19:61–109. https://doi.org/10.1016/s0197-2456(97)00078-0 PMID:9492970

24. Anderson GL, Manson J, Wallace R, Lund B, Hall D, Davis S, Shumaker S, Wang CY, Stein E, Prentice RL. Implementation of the women's health initiative study design. Ann Epidemiol. 2003; 13:S5–17. https://doi.org/10.1016/s1047-2797(03)00043-7 PMID:14575938

25. Leek JT, Johnson WE, Parker HS, Jaffe AE, Storey JD. The sva package for removing batch effects and other unwanted variation in high-throughput experiments. Bioinformatics. 2012; 28:882–83. https://doi.org/10.1093/bioinformatics/bts034 PMID:22257669

26. Houseman EA, Accomando WP, Koestler DC, Christensen BC, Marsit CJ, Nelson HH, Wiencke JK, Kelsey KT. DNA methylation arrays as surrogate measures of cell mixture distribution. BMC Bioinformatics. 2012; 13:86. https://doi.org/10.1186/1471-2105-13-86 PMID:22568884
