# Supplementary material for: Blood DNA methylation sites predict death risk in a longitudinal study of 12,300 individuals
Source: Aging (Albany NY). 2020 Jul 22;12(14):14092–124. doi: 10.18632/aging.103408 (PMC7425458; doi:10.18632/aging.103408)
Supplement: Supplementary Figures [file aging-12-103408-s009..pdf]

SUPPLEMENTARY FIGURES

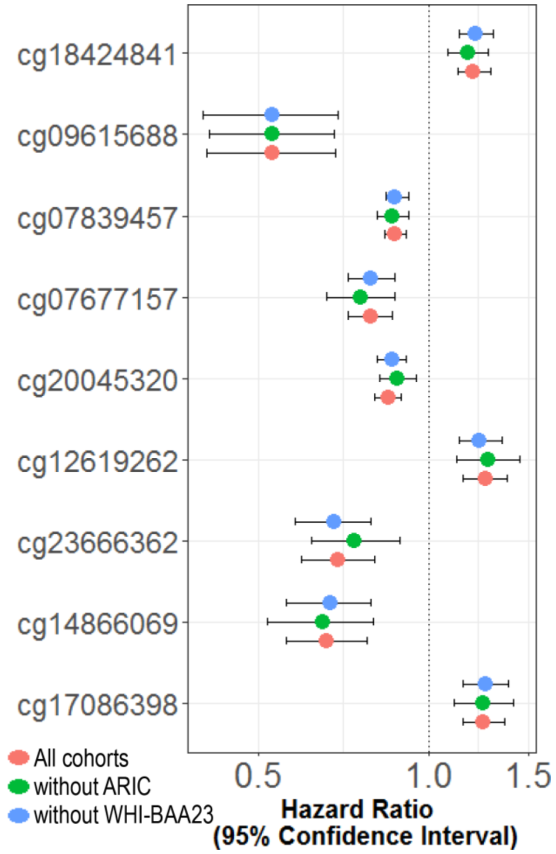

Supplementary Figure 1. Sensitivity analysis comparing hazard ratios of the fully-adjusted meta-analysis, including all cohorts, all excluding ARIC, or all excluding WHI-BAA23.

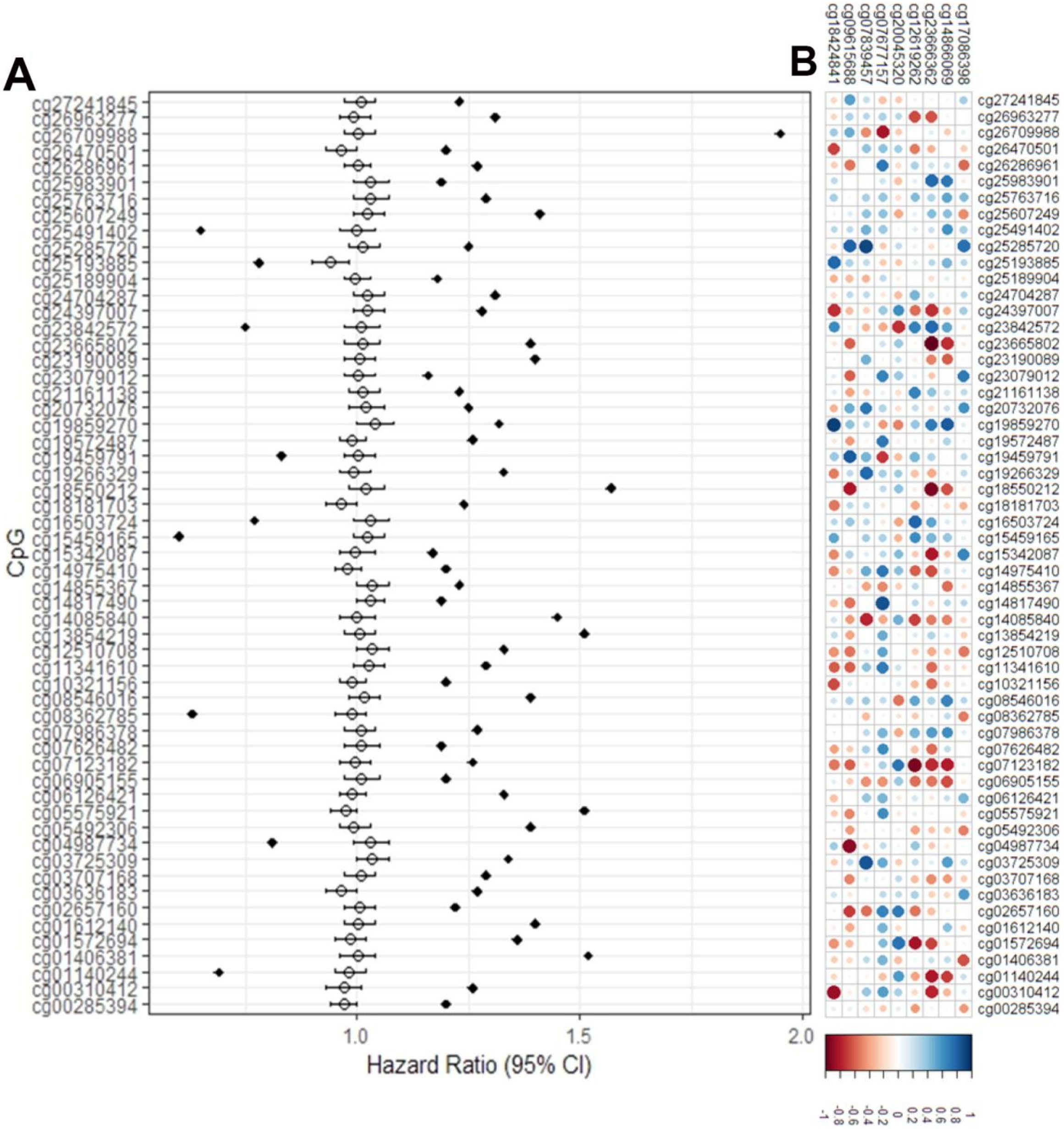

Supplementary Figure 2. (**A**) All-cause mortality association of 57 out of 58 CpGs identified with mortality in Zhang et al. (black dots) and all-cause mortality association of the same CpGs in the pooled meta-analysis (white dots with 95% confidence intervals). (**B**) Association of methylation levels of 57 out of 58 CpGs identified with mortality in Zhang et al. and our FDR-significant CpGs in all cohorts.

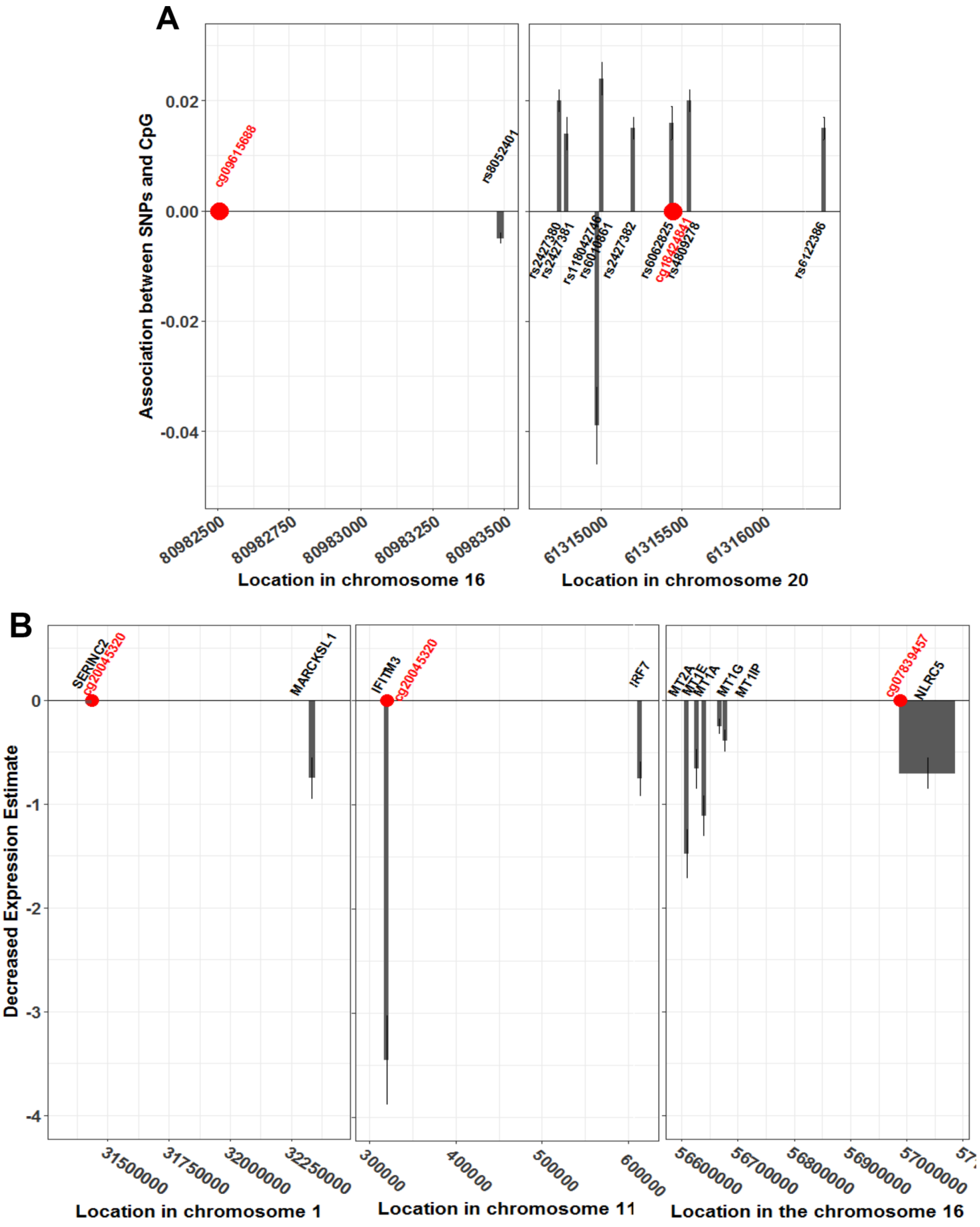

Supplementary Figure 3. (**A**) Methylation quantitative trait loci (meQTL) analysis and (**B**) expression quantitative quantitative trait methylation (eQTM) analysis in KORA.
