# Supplementary material for: Blood DNA methylation sites predict death risk in a longitudinal study of 12,300 individuals
Source: Aging (Albany NY). 2020 Jul 22;12(14):14092–124. doi: 10.18632/aging.103408 (PMC7425458; doi:10.18632/aging.103408)
Supplement: Supplementary Tables 3-5, 7-9, 11, 13-16, 18-22 [file aging-12-103408-s008..pdf]

SUPPLEMENTARY TABLES

Supplementary Table 3. CpG loci where blood DNA methylation was associated (FDR<0.05) with all-cause mortality in the fixed-effect meta-analysis from the fully adjusted model.

Probe name	CHR	Distance to nearest gene (bp)	Nearest gene (10 Mp)[a]	Gene group	Relation to CpG Island	HR[b]	95% CI	*p*	Mean methylation level	Bonferroni significance	FDR-significant in basic model	Methylation level (Mean±SD)
cg17086398	1	0	*SERINC2*	Body		1.25	(1.15;1.36)	4.86E-07	0.29		1	0.29 ± 0.06
cg14866069	4	579	*BMPR1B*			0.66	(0.56;0.78)	4.85E-07	0.85			0.85 ± 0.05
cg23666362	4	516	*MIR1973*	TSS1500		0.69	(0.59;0.8)	8.00E-07	0.82			0.81 ± 0.04
cg12619262	7	6276	*CHST12*			1.26	(1.16;1.37)	1.76E-07	0.75			0.75 ± 0.07
cg20045320	11	116	*IFITM3*		S_Shore	0.85	(0.8;0.9)	4.06E-09	0.54	1	1	0.54 ± 0.09
cg07677157	12		NA[a]			0.79	(0.72;0.86)	2.00E-07	0.16		1	0.18 ± 0.06
cg07839457	16	435	*NLRC5*	TSS1500	N_Shore	0.87	(0.84;0.91)	2.40E-09	0.46	1	1	0.45 ± 0.11
cg09615688	16		NA[a]			0.53	(0.41;0.68)	9.32E-07	0.91		1	0.90 ± 0.03
cg18424841	20		NA[a]		Island	1.2	(1.13;1.28)	2.80E-08	0.7	1		0.69 ± 0.09

[a]Nearest gene was far more than 10 Mp.
[b]Effect estimates represent hazard ratio per 10% increase in DNA methylation. CHR = chromosome; HR = hazard ratio; 95% CI = 95% confidence interval; p = p-value; SD = standard deviation.

Supplementary Table 4. Hazard ratios for FDR-significant fully-adjusted CpGs in basic and fully adjusted models CHR = chromosome; HR = hazard ratio; 95% CI = 95% confidence interval; p = p-value; SD = standard deviation.

Probe name	CHR	Fully adjusted model			Basic model		
		HR	95% CI	*p*	HR	95% CI	*p*
cg17086398	1	1.25	(1.15; 1.36)	4.86E-07	1.37	(1.28; 1.47)	5.32E-20
cg14866069	4	0.66	(0.56; 0.78)	4.85E-07	0.84	(0.75; 0.94)	2.21E-03
cg23666362	4	0.69	(0.59; 0.8)	8.04E-07	0.81	(0.72; 0.9)	2.02E-04
cg12619262	7	1.26	(1.15; 1.38)	1.76E-07	1.13	(1.05; 1.21)	6.77E-04
cg20045320	11	0.85	(0.80; 0.90)	4.06E-09	0.82	(0.78; 0.86)	2.61E-16
cg07677157	12	0.79	(0.72; 0.86)	2.00E-07	0.78	(0.72; 0.84)	1.31E-10
cg07839457	16	0.87	(0.84; 0.91)	2.40E-09	0.88	(0.85; 0.92)	3.40E-11
cg09615688	16	0.53	(0.41; 0.68)	9.32E-07	0.60	(0.51; 0.72)	8.96E-09
cg18424841	20	1.20	(1.13; 1.28)	2.80E-08	1.10	(1.05; 1.15)	2.00E-04

Supplementary Table 5. Summary of models.

Cohorts	Model used	Maximum # probes considered	Basic model		Fully adjusted model	
			Lambda	FDR-significant CpGs	Lambda	FDR-significant CpGs
ARIC	Cox Regression	406712	2.46	226	1.63	5
FHS Study 1	Cox Regression[b]	417934	0.94	3	1.04	0
FHS Study 2	Cox Regression[b]	407580	1.32	17	1.16	1
InChianti	Cox Regression	407179	1.41	0	1.44	0
KORA	Cox Regression[a]	330133	0.96	4	0.94	0
LBC 1921	Cox Regression	393529	1.15	0	1.22	0
LBC 1936	Cox Regression	385450	1.04	0	1	0
NAS	Cox Regression	395005	0.86	0	0.9	0
TwinsUK	Cox Regression [a,c]	426,120	1.02	0	0.98	0
WHI-BAA23	Cox Regression	419176	1.7	14	2.02	1
WHI-EMPC-EA	Cox Regression[d]	250537	1.23	9	0.93	0
WHI-EMPC-AA	Cox Regression[d]	218018	0.92	0	1.09	0
All cohorts	Fixed effect meta-analysis[e]	426724	1.12	257	0.94	9

[a]Cohort used as predictor of residuals from linear regression analysis between each probe and sets of covariates.
[b]Cohort included cluster for family structure.
[c]Cohort included random intercepts for zygosity and family structure.
[d]Cohort considered only CpGs with coefficient of variation >5%.
[e]Analysis of each CpG site included results of at least three cohorts.
Basic model: adjusted for age (categories), gender, technical variables, white blood cell count.
Fully adjusted model: adjusted for age (categories), gender, technical variables, white blood cell count, education level, physical activity, smoking status, smoking consumption (packyears), body mass index (categories), alcohol consumption, prior coronary heart disease (y/n), diabetes (y/n), hypertension (y/n), cancer (y/n).

Supplementary Table 7. I^2 measure of heterogeneity from random-effect meta-analysis in each FDR-significant fully-adjusted CpG.

Probe name	CHR	Distance to nearest gene (bp)	Nearest gene (10 Mp)[a]	Gene group	Relation to CpG island	I^2
cg17086398	1	0	*SERINC2*	Body		0.02
cg14866069	4	579	*BMPR1B*			0
cg23666362	4	516	*MIR1973*	TSS1500		0.01
cg12619262	7	6276	*CHST12*			0.01
cg20045320	11	116	*IFITM3*		S_Shore	53.53
cg07677157	12		NA[a]			0
cg07839457	16	435	*NLRC5*	TSS1500	N_Shore	0
cg09615688	16		NA[a]			29.9
cg18424841	20		NA[a]		Island	2.65

[a]Nearest gene was far more than 10 Mp.
[b]Effect estimates represent hazard ratio per 10% increase in DNA methylation.
CHR = chromosome; HR = hazard ratio; 95% CI = 95% confidence interval; p = p-value; SD = standard deviation.

Supplementary Table 8. FDR-significant fully-adjusted CpGs in fixed effect meta-analysis with exclusion of ARIC.

Probe name	CHR	Distance to nearest gene (bp)	Nearest gene (10 Mp)[a]	Gene group	Relation to CpG island	HR	95% CI	*p*	Mean DNA methylation level
cg17086398	1	0	*SERINC2*	Body		1.25	(1.11; 1.41)	3.80E-04	0.29
cg14866069	4	579	*BMPR1B*			0.65	(0.52; 0.8)	6.86E-05	0.84
cg23666362	4	516	*MIR1973*	TSS1500		0.74	(0.62; 0.89)	1.35E-03	0.8
cg12619262	7	6276	*CHST12*			1.27	(1.12; 1.45)	3.31E-04	0.74
cg20045320	11	116	*IFITM3*		S_Shore	0.88	(0.82; 0.95)	8.76E-04	0.54
cg07677157	12		NA[a]			0.76	(0.66; 0.87)	4.78E-05	0.19
cg07839457	16	435	*NLRC5*	TSS1500	N_Shore	0.86	(0.81; 0.92)	3.70E-06	0.45
cg09615688	16		NA[a]			0.53	(0.41; 0.68)	9.32E-07	0.89
cg18424841	20		NA[a]		Island	1.17	(1.08; 1.27)	1.90E-04	0.68

[a]Nearest gene was far more than 10 Mp.
[b]Effect estimates represent hazard ratio per 10% increase in DNA methylation.
CHR = chromosome; HR = hazard ratio; 95% CI = 95% confidence interval; p = p-value; SD = standard deviation.

Supplementary Table 9. FDR-significant fully-adjusted CpGs in fixed effect meta-analysis with exclusion of WHI-Study 1.

Probe name	CHR	Distance to nearest gene (bp)	Nearest gene (10 Mp)[a]	Gene group	Relation to CpG island	HR	95% CI	*p*	Mean DNA methylation level
cg17086398	1	0	*SERINC2*	Body		1.26	(1.15; 1.38)	4.30E-07	0.29
cg14866069	4	579	*BMPR1B*			0.67	(0.56; 0.79)	2.23E-06	0.85
cg23666362	4	516	*MIR1973*	TSS1500		0.68	(0.58; 0.79)	1.16E-06	0.81
cg12619262	7	6276	*CHST12*			1.23	(1.13; 1.35)	5.93E-06	0.74
cg20045320	11	116	*IFITM3*		S_Shore	0.86	(0.81; 0.91)	2.94E-07	0.54
cg07677157	12		NA[a]			0.79	(0.72; 0.87)	2.57E-06	0.17
cg07839457	16	435	*NLRC5*	TSS1500	N_Shore	0.87	(0.84; 0.92)	1.30E-08	0.45
cg09615688	16		NA[a]			0.53	(0.4; 0.69)	2.57E-06	0.9
cg18424841	20		NA[a]		Island	1.21	(1.13; 1.3)	1.53E-08	0.69

[a]Nearest gene was far more than 10 Mp.
[b]Effect estimates represent hazard ratio per 10% increase in DNA methylation.
CHR = chromosome; HR = hazard ratio; 95% CI = 95% confidence interval; p = p-value; SD = standard deviation.

Supplementary Table 11. Miettinen's population attributable factor for NAS, WHI-EMPC-EA, and WHI-EMPC-AA as well as weighted combination (average).

CpG	NAS	WHI-EMPC-EA	WHI-EMPC-AA	Mean	SD
cg17086398	6.47	5.55	3.57	5.20	1.21
cg14866069	-17.20	-6.77	-20.09	-14.69	5.72
cg23666362	-15.19	.	.	-15.19	0.00
cg12619262	7.33	-1.18	4.27	3.48	3.52
cg20045320	-5.34	-0.67	-1.42	-2.48	2.05
cg07677157	-7.29	-9.43	-27.86	-14.86	9.23
cg07839457	-4.08	-3.07	-3.43	-3.53	0.42
cg09615688	.	1.43	.	1.43	0.00
cg18424841	5.97	3.51	4.91	4.80	1.01

Supplementary Table 13. Standardized betas identifying the linear relationship between FDR-significant fully-adjusted CpGs and epigenetic aging clock in NAS, after adjusting all conventional risk factors.

Epigenetic age	cg17086398	cg14866069	cg23666362	cg12619262	cg20045320	cg07677157	cg07839457	cg18424841
	Est (p)	Est (p)	Est (p)	Est (p)	Est (p)	Est (p)	Est (p)	Est (p)
Horvath epigenetic aging clock (years)	-0.09(0.1)	0.03(0.51)	0.01(0.78)	0.23(0)	-0.27(0)	-0.13(0.02)	-0.19(0)	0.11(0.03)
Hannum epigenetic aging clock (years)	-0.07(0.24)	-0.17(0)	-0.14(0.01)	0.36(0)	-0.08(0.23)	-0.06(0.34)	-0.28(0)	0.18(0)
Weidener epigenetic aging clock (years)	0.02(0.69)	-0.03(0.39)	-0.01(0.72)	0.08(0.1)	-0.07(0.11)	-0.04(0.46)	-0.04(0.41)	0.06(0.16)
PhenoAge (years)	-0.04 (0.5)	-0.11 (0.01)	-0.11 (0.02)	0.02 (0.76)	-0.27 (0)	0 (0.93)	-0.19 (0)	0.13 (0.01)
Mortality risk score	0.04 (0.44)	-0.22 (0)	-0.19 (0)	0.34 (0)	-0.19 (0)	-0.49 (0)	-0.03 (0.57)	-0.07 (0.22)

Est = estimate; p = p-value.

Supplementary Table 14. Association with all-cause mortality and DNA methylation levels at FDR-significant CpGs, adjusting for epigenetic acceleration ages in the Normative Aging Study (NAS).

Association with mortality	CpG alone	CpG + DNAmAge acceleration		CpG + PhenoAge acceleration	
		CpG	DNAmAge acceleration	CpG	PhenoAge acceleration
	HR (95% CI)	HR (95% CI)	HR (95% CI)	HR (95% CI)	HR (95% CI)
cg17086398	1.03 (0.83-1.27)	1.02 (0.82-1.27)	0.99 (0.97-1.03)	1.02 (0.82-1.26)	1.01 (0.98-1.03)
cg14866069	0.44 (0.30-0.70)	0.44 (0.30-0.69)	0.99 (0.97-1.02)	0.44 (0.28-0.70)	1.00 (0.98-1.03)
cg23666362	0.80 (0.52-1.23)	0.80 (0.52-1.23)	1.00 (0.97-1.03)	0.78 (0.50-1.20)	1.01 (0.99-1.03)
cg12619262	1.08 (0.88-1.33)	1.09 (0.89-1.34)	0.99 (0.97-1.02)	1.09 (0.88-1.33)	1.01 (0.98-1.03)
cg20045320	0.94 (0.81-1.09)	0.93 (0.81-1.08)	0.99 (0.97-1.02)	0.94 (0.82-1.09)	1.01 (0.98-1.03)
cg07677157	0.70 (0.52-0.94)	0.70 (0.52-0.94)	0.99 (0.97-1.02)	0.70 (0.52-0.94)	1.00 (0.98-1.03)
cg07839457	0.87 (0.78-0.97)	0.86 (0.77-0.96)	0.99 (0.97-1.02)	0.87 (0.78-0.97)	1.01 (0.98-1.03)
cg18424841	1.09 (0.94-1.26)	1.07 (0.92-1.25)	1.00 (0.97-1.02)	1.09 (0.94-1.26)	1.01 (0.98-1.03)

Supplementary Table 15. Association with all-cause mortality and DNA methylation levels at FDR-significant CpGs adjusting for mortality risk score in the Normative Aging Study (NAS).

Association with mortality	CpG alone	CpG + mortality risk score	
		CpG	Mortality risk score
	HR (95% CI)	HR (95% CI)	HR (95% CI)
cg17086398	1.03 (0.83-1.27)	1.12 (0.89-1.41)	1.68 (1.15-2.47)
cg14866069	0.44 (0.30-0.70)	0.45 (0.28-0.74)	1.39 (0.95-2.04)
cg23666362	0.80 (0.52-1.23)	0.87 (0.55-1.36)	1.55 (1.06-2.27)
cg12619262	1.08 (0.88-1.33)	1.01 (0.81 – 1.26)	1.60 (1.08-2.35)
cg20045320	0.94 (0.81-1.09)	0.96 (0.82-1.12)	1.57 (1.08-2.28)
cg07677157	0.70 (0.52-0.94)	0.74 (0.55-1.01)	1.46 (1.00-2.14)
cg07839457	0.87 (0.78-0.97)	0.88 (0.79-0.99)	1.51 (1.05-2.18)
cg18424841	1.09 (0.94-1.26)	1.07 (0.92-1.24)	1.59 (1.10-2.30)

Supplementary Table 16. Enrichment analysis for genes identified in GWAS of death-related factors.

Disease	Gene	Enrichment p-value	Enrichment_FDR
Alcohol dependence	*SERINC2*	0.002	0.004
HDL cholesterol	*NLRC5*	0.022	0.022

p = p-value; FDR = false discovery rate.

Supplementary Table 18. Pathways analysis with DAVID.

Gene	Official gene name	Diseases	Disease class	*p*
NLRC5	NLR family CARD domain containing 5	Chronic renal failure, kidney failure, chronic coronary disease, erythrocyte count, type 2 diabetes	CARDIOVASCULAR, HEMATOLOGICAL, METABOLIC, RENAL	>0.05
BMPR1B	Bone morphogenetic protein receptor type 1B	Alcoholism, attention deficit disorder with hyperactivity, bone mineral density, cleft lip, cleft palate, hypertension, increased ovulation rate, juvenile polyposis, obesity, premature ovarian failure, polycystic ovarian syndrome, primary ovarian insufficiency, puberty (delayed), puberty (precocious), thrombophilia, tobacco use disorder	CARDIOVASCULAR, CHEMDEPENDENCY, DEVELOPMENTAL, METABOLIC, OTHER, PSYCH, REPRODUCTION	>0.05
CHST12	Carbohydrate sulfotransferase 12	Malaria, placenta diseases, pregnancy complications, parasitic	INFECTION	>0.05
IFITM3	Interferon induced transmembrane protein 3	Ulcerative colitis	IMMUNE	>0.05

Supplementary Table 19. Association between fully-adjusted FDR-significant CpGs and SNPs (meQTL analysis) in KORA.

Fully-adjusted FDR-significant CpGs			SNP			Est.	SE	*p*
Name	CHR	Position	Name	CHR	Position			
cg09615688	16	80982506	rs8052401	16	80983487	-0.005	0.001	3.81E-08
cg18424841	20	61315444	rs2427380	20	61314740	0.020	0.002	2.27E-16
cg18424841	20	61315444	rs2427381	20	61314785	0.014	0.003	2.50E-08
cg18424841	20	61315444	rs118042746	20	61314972	-0.039	0.007	2.47E-09
cg18424841	20	61315444	rs6010861	20	61315002	0.024	0.003	9.67E-17
cg18424841	20	61315444	rs2427382	20	61315199	0.015	0.002	6.91E-10
cg18424841	20	61315444	rs6062825	20	61315436	0.016	0.003	2.37E-06
cg18424841	20	61315444	rs4809278	20	61315545	0.020	0.002	6.81E-16
cg18424841	20	61315444	rs6122386	20	61316386	0.015	0.002	6.20E-10

CHR = chromosome; Est = estimate; SE = standard error; p = p-value.

Supplementary Table 20. Association between fully-adjusted FDR-significant CpGs and gene expression (eQTM analysis) in KORA.

Probe name	CHR	Distance to nearest gene (bp)	Nearest gene	Influenced gene name	Est	SE	*p*	FDR
cg17086398	1	0	*SERINC2*	*MARCKSL1*	-0.75	0.20	1.77E-04	5.92E-03
cg20045320	11	116	*IFITM3*	*IFITM3*	-3.45	0.43	3.19E-15	7.48E-13
cg20045320	11	116	*IFITM3*	*IRF7*	-0.76	0.17	8.78E-06	4.11E-04
cg07839457	16	435	*NLRC5*	*MT2A*	-1.48	0.24	7.92E-10	9.27E-08
cg07839457	16	435	*NLRC5*	*MT1E*	-0.66	0.19	5.17E-04	1.34E-02
cg07839457	16	435	*NLRC5*	*MT1A*	-1.11	0.20	1.98E-08	1.54E-06
cg07839457	16	435	*NLRC5*	*MT1G*	-0.25	0.07	6.70E-04	1.57E-02
cg07839457	16	435	*NLRC5*	*MT1IP*	-0.39	0.10	1.64E-04	5.92E-03
cg07839457	16	435	*NLRC5*	*NLRC5*	-0.70	0.15	3.67E-06	2.15E-04

CHR = chromosome; Est = estimate; SE = standard error; p = p-value; FDR = false discovery rate.

Supplementary Table 21. Causal association between coronary heart disease, kidney function (serum creatinine), and methylation at FDR-significant CpGs in KORA and ARIES.

Disease	Methylation locus	OR	95% LCI	95% UCI	P	methQTL cohort	N SNPs	MR method
Coronary heart disease	cg09615688	1.508	1.0199	2.2297	0.0395	KORA	1	Wald ratio
	cg18424841	1.0058	0.9994	1.0122	0.0743	ARIES	1	Wald ratio
	cg18424841	0.8506	0.7292	0.9922	0.0944	KORA	7	MR Egger
	cg18424841	1.01	0.9218	1.1068	0.8375	KORA	7	Weighted mode
	cg18424841	1.0007	0.9399	1.0656	0.9814	KORA	7	Weighted median
Serum creatinine	cg09615688	0.8816	0.7449	1.0435	0.1429	KORA	1	Wald ratio
	cg18424841	1.0014	0.9989	1.0039	0.2771	ARIES	1	Wald ratio
	cg18424841	0.9279	0.8297	1.0377	0.4148	KORA	3	MR Egger
	cg18424841	0.9903	0.957	1.0246	0.6305	KORA	3	Weighted mode
	cg18424841	0.9939	0.9637	1.025	0.6969	KORA	3	Weighted median

Odds ratio (OR), lower 95% confidence interval (LCI), and upper 95% confidence interval (UCI) given per 10% higher methylation. Associations for coronary heart disease taken from (PMC4589895) and association for serum creatinine taken from (PMC4735748). Associations for ARIES methQTLs extracted from MR-base (PMC5976434) using middle age estimates for methQTLs from ARIES cohort (PMC4818469). MR = mendelian randomization; N SNPs = number of SNPs (instruments) used for the MR analyses; P = p-value.

Supplementary Table 22. Association of neutrophil–lymphocyte ratio (NLR) with all-cause mortality, with and without adjustment for cell type proportion in Normative Aging Study.

NLR association with mortality	Without adjusting for cell proportions		Adjusting for cell proportions	
	HR (95% CI)	*p*	HR (95% CI)	*p*
without any CpG inclusion	1.08 (1.00 – 1.17)	0.04	1.06 (0.92 – 1.21)	0.43
cg17086398	1.08 (1.00 – 1.17)	0.045	1.06 (0.93 – 1.21)	0.41
cg14866069	1.13 (1.05 – 1.22)	0.002	1.03 (0.90 – 1.18)	0.68
cg23666362	1.10 (1.02 – 1.19)	0.017	1.04 (0.90 – 1.20)	0.61
cg12619262	1.08 (1.00 – 1.17)	0.042	1.05 (0.92 – 1.21)	0.45
cg20045320	1.08 (1.00 – 1.17)	0.042	1.05 (0.92 – 1.20)	0.46
cg07677157	1.09 (1.01 – 1.18)	0.034	1.06 (0.93 – 1.21)	0.39
cg07839457	1.07 (0.99 – 1.15)	0.06	1.00 (0.88 – 1.15)	0.97
cg18424841	1.10 (1.02 – 1.19)	0.02	1.06 (0.93 – 1.21)	0.38
